# Mechanochemical synthesis of unsymmetrical salens for the preparation of Co–salen complexes and their evaluation as catalysts for the synthesis of α-aryloxy alcohols via asymmetric phenolic kinetic resolution of terminal epoxides

**DOI:** 10.3762/bjoc.18.147

**Published:** 2022-10-10

**Authors:** Shengli Zuo, Shuxiang Zheng, Jianjun Liu, Ang Zuo

**Affiliations:** 1 State Key Laboratory of Chemical Resource Engineering, Department of Applied Chemistry, College of Chemistry, Beijing University of Chemical Technology, Beijing 100029, China; 2 Department of Pharmaceutical Sciences, College of Pharmacy and UICentre, University of Illinois at Chicago, Chicago, Illinois 60612, United States

**Keywords:** α-aryloxy alcohols, chiral Co–salen, HKR, mechanochemistry, phenolic KR

## Abstract

In this paper, we report the mechanochemical synthesis of unsymmetrical salens using grinding and ball milling technologies, respectively, both of which were afforded in good yield. The chelating effect of the unsymmetrical salens with zinc, copper, and cobalt was studied and the chiral Co–salen complex **2f** was obtained in 98% yield. Hydrolytic kinetic resolution (HKR) of epichlorohydrin with water catalyzed by complex **2f** (0.5 mol %) was explored and resulted in 98% ee, suggesting complex **2f** could serve as an enantioselective catalyst for the asymmetric ring opening of terminal epoxides by phenols. A library of α-aryloxy alcohols **3** was thereafter synthesized in good yield and high ee using **2f** via the phenolic KR of epichlorohydrin.

## Introduction

In the past decade, more than twenty chiral small molecule drugs were approved by the FDA, including ruxolitinib, afatinib, sonidegib, encorafenib, lorlatinib, darolutamide, alpelisib, artesunate, maribavir, ponesimod, daridorexant and others [1–3]. The enantioselective synthesis in modern chemistry turns out to be accumulatively essential for the preparation of chiral drugs, which is a huge growing market in the future. Indeed, the asymmetric ring opening of terminal epoxides is one of the most important strategies for synthesizing drug-like building blocks and key organic intermediates in the drug discovery and process chemistry [4–6]. Chiral metal–salen complexes were designed for catalyzing reaction processes that resulted in good yield, high regioselective and enantioselective control for the asymmetric ring opening of terminal epoxides. Various metals have been explored to optimize the catalytic properties of chiral metal–salens, such as Cr [7], Co [8], Fe [9], Ti [10], Al [11], Y [12], and Mn [13] and investigated with numerous nucleophiles to afford chiral molecules. In addition to the variation of metals, salen ligands have also been studied with regard to conformational differences, for instance, oligosalen [14], macrocyclic oligosalen [15], and polymeric salen [16].

**Figure 1:**
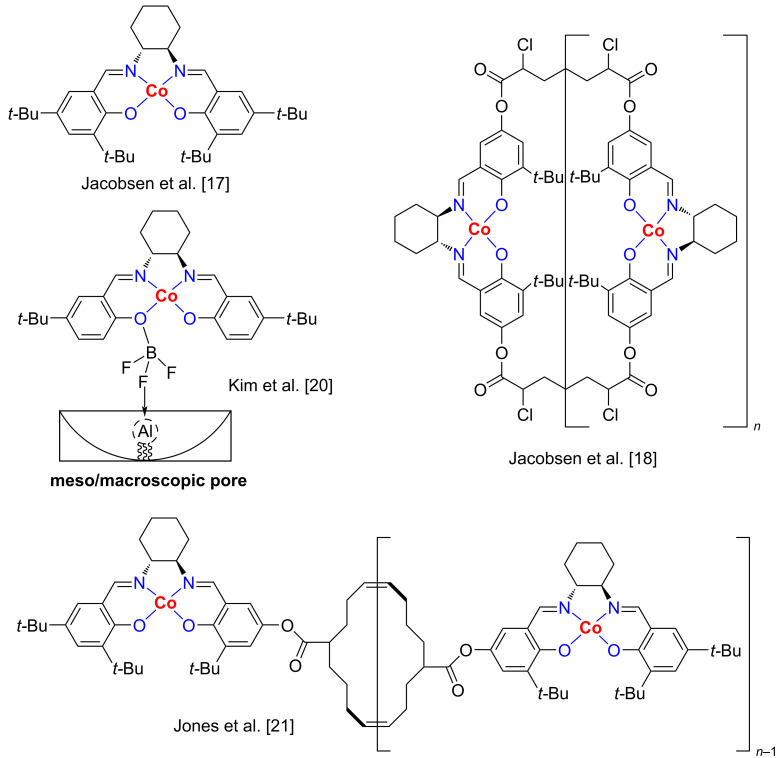
Representative asymmetric Co–salen catalysts.

Jacobsen and co-workers reported the first synthesis of α-aryloxy alcohols through the phenolic kinetic resolution (KR) of terminal epoxides using a Co–salen catalyst [17]. Since their discovery, researchers have investigated several Co–salen complexes for the KR of epoxides with phenols as nucleophiles (Figure 1) [18–19]. Kim et al. described a catalytic system of a chiral Co–salen immobilized on meso/macroporous silica monoliths for the ring opening of epoxides [20]. Jones et al. designed a cyclooctene-based Co–salen macrocycle catalyst for the phenolic KR of epichlorohydrin and 1,2-epoxyhexane [21]. However, these Co–salen systems suffer from several limitations such as tedious preparation of salen scaffolds, excess use of epoxides, high catalyst loadings, narrow scope and the need of Lewis acidic or basic co-catalysts [22–24]. A more efficient preparation of Co–salen catalysts is therefore of a great need for the asymmetric ring opening of epoxides, and thus became extremely attractive to us.

**Scheme 1:**
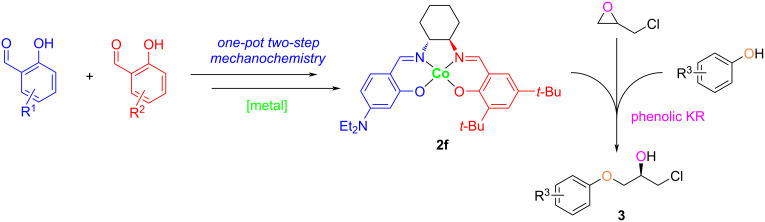
Synthetic approach to our unsymmetrical Co–salen catalyst **2f** for the asymmetric synthesis of α-aryloxy alcohols.

The synthesis of novel Co–salen catalysts begins with the design and preparation of suitable salen compounds, sometimes are described as bis-imine Schiff bases. Imines were originally synthesized by Schiff from the condensation of carbonyls with amines [25]. Thereafter, syntheses of salens were extensively reported using timely technologies [26–29]. Inspired by the mechanochemical chemistry technology to simplify chemical processes and eliminate the use of organic solvents, salen compounds have been synthesized by the “green” grinding strategy previously [30–39]. Herein, we report a one-pot two-step mechanochemical synthesis of unsymmetrical salens for the preparation of Co–salen complexes and their evaluation as catalyst for the synthesis of α-aryloxy alcohols through the phenolic KR of terminal epoxides (Scheme 1). Indeed, advantages to break the *C*_2_-symmetry in Co–salen complexes were reported before [23,40]. In addition, a Lewis basic NEt_2_ (‒N(CH_2_CH_3_)_2_) group was introduced to the salen scaffold to facilitate purification, enhance catalytic efficiency, and improve the thermal stability, as was shown in the synthesis of fluorescent probes [41–42]. The chelating effect of salen compounds **1** with different metals were explored as well. Furthermore, we present the hydrolytic kinetic resolution (HKR) of epichlorohydrin with water using Co–salen complexes **2**, and α-aryloxy alcohols were synthesized by the **2f** catalytic system through the asymmetric ring opening of epichlorohydrin and phenols.

## Results and Discussion

**Scheme 2:**
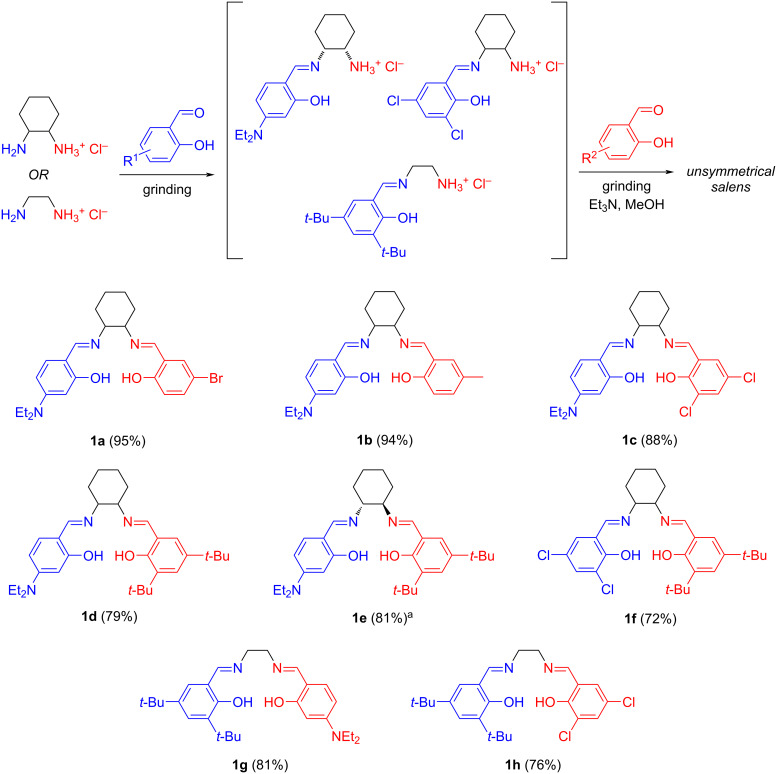
Mechanochemical one-pot two-step synthesis of unsymmetrical salens **1a**–**h**. Reaction conditions: salicylaldehyde (1 mmol) and diamine unilateral hydrochloride salt (2 mmol) were grinded in an agate mortar for 10 min. Then, triethylamine (4 mmol), methanol (0.12 μL/mg), and the second salicylaldehyde (1 mmol) were added to the mortar/pestle, and the mixture was grinded for further 20 min. The products were purified by column chromatography using *n*-hexane/ethyl acetate as the eluent. ^a^Starting material was *trans*-1,2-diaminocyclohexane monohydrochloride.

The mechanochemical study examined the synthesis of several unsymmetrical salens using monoammonium salts and salicylaldehydes (Scheme 2). Agate mortar and pestle were used for the one-pot two-step mechanochemical reactions (see Supporting Information File 1). Initially, 1,2-diaminocyclohexane or ethylenediamine monohydrochlorides were grinded with a half equivalent of 4-diethylamino (Et_2_N‒), 3,5-dichloro (Cl‒), or 3,5-di-*tert*-butyl (*t*-Bu‒) salicylaldehydes (blue moieties in Scheme 2) for 10 minutes. The synthesis of diamine monohydrochlorides and characterization data of mono-imine ammonium salts were described before [30–33,36]. This process generates mono-imine ammonium salts as the stable intermediates in the mortar. Without implementing treatment such as filtration, evaporation of solvents, or further purification, mono-imine ammonium salts were subsequently treated with triethylamine (Et_3_N), half equivalent of 5-bromo (Br‒), 5-methyl, 4-diethylamino (Et_2_N‒), 3,5-dichloro (Cl‒), or 3,5-di-*tert*-butyl (*t*-Bu‒) salicylaldehydes (red moieties in Scheme 2), and trace methanol, followed by grinding for 20 minutes for the second reaction step to complete, monitored by TLC. A trace amount of methanol was used to lubricate the molecular surface for an improved performance (known as liquid-assisted grinding, LAG) [42]. Unsymmetrical salens **1a**‒**h** were obtained in the yield of 72% to 95% after being purified by column chromatography. Bromo-containing salen **1a** was yielded the best (95%), presumably due to the strong electron-withdrawing effect of bromine, enhancing the electrophilic property of bromo-substituted salicylaldehyde. Because of the poor solubility in the eluent, the yield of dichloro-containing **1c** (88%) was lower than **1a** after isolating by column chromatography. This was also found between **1g** (81%) and **1h** (76%). Yields of **1d** (79%), **1e** (81%), and **1f** (72%) were less than **1a**‒**c**, caused by the steric hindrance of di-*tert*-butyl groups. In the aspect of characterization of salens, two singlets were shown at around 8 ppm in the ^1^H NMR spectrum, indicating two unsymmetrical imines. The broad peak at around 13 ppm was assigned to the phenolic OH groups. The signal at around 1615 cm^−1^ in the IR spectrum could also indicate the formation of imine (see Supporting Information File 1).

In addition to the use of grinding technology, a self-made ball mill was applied to the synthesis of unsymmetrical salens by us. The method and its principle were described previously [43–45]. Ball mill systems have several advantages including superior mixing, continuous operation, and enclosed reaction environment. Our ball mill system was designed to mount a 40 mL glass reactor with zirconia and/or alumina composite balls (3.20 mm and 2.16 mm in diameter, respectively). Considering the safety in the synthesis of unsymmetrical salens, the working speed was set to be 700 rev/min. Similarly to the above reaction conditions, amounts of chemicals and workup, the first reaction step between amino monohydrochlorides and salicylaldehydes (blue in Scheme 2) took 1 hour for reaction completion. After adding another salicylaldehyde (red in Scheme 2), Et_3_N, and methanol, the second reaction step was completed in an additional hour, monitored by TLC. Yields of unsymmetrical salens using grinding and ball milling were summarized in Table 1. We were surprised that the overall yield from ball milling was lower than the overall yield from grinding, suggesting a higher revolution per minute (RPM) could be necessary to increase the reaction yield using ball milling. It is assumed that the forces are not equivalent in both techniques and probably pressure-induced activation and shearing deformation of reactant particles are more efficient using the grinding.

**Table 1:** Yields of unsymmetrical salens **1** using grinding and ball milling.

Entry	ID	Grinding/yield (%)	Ball milling/yield (%)

1	**1a**	95	82
2	**1b**	94	71
3	**1c**	88	77
4	**1d**	79	66
5	**1e**	81	68
6	**1f**	72	57
7	**1g**	81	72
8	**1h**	76	61

**Scheme 3:**
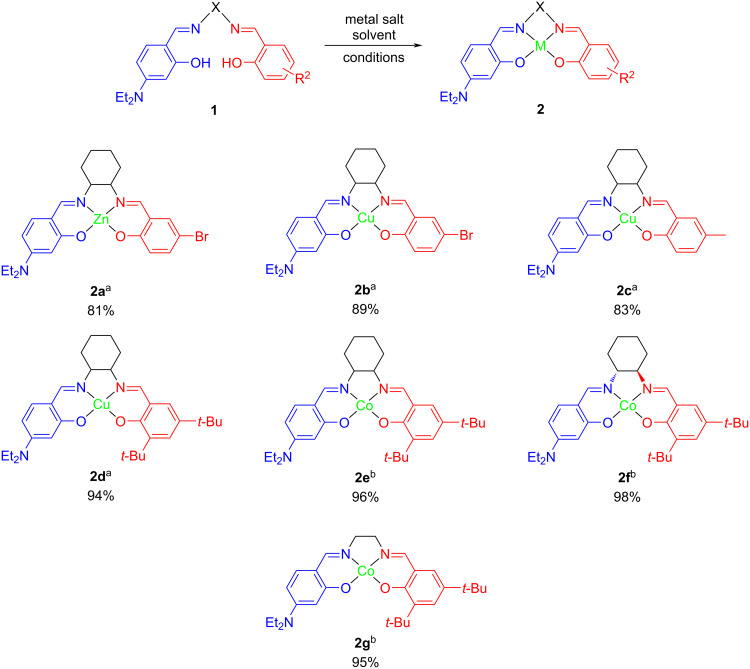
Synthesis of unsymmetrical metal–salen complexes **2**. Reaction conditions a: metal acetate hydrate (1 mmol) and MeOH (12 mL) were dropwise added to compound **1** (1 mmol) in EtOH (7 mL) in a round-bottomed flask, and refluxed for 4 hours under nitrogen gas. Products were afforded by filtration and washed with cold methanol (20 mL × 2); Reaction conditions b: ligand **1** (1 mmol), cobalt(II) acetate tetrahydrate (1.2 mmol), and MeOH (10 mL) were gradually added to a round-bottomed flask, and stirred at 0 °C for 40 min under nitrogen gas. Products were isolated by filtration and washed with cold methanol (2 × 20 mL).

We next examined the chelating effect of the above salens **1** with different transition metals. A library of metal–salen complexes was synthesized as outlined in Scheme 3. Reaction conditions were described previously [17,46]. For reactions using Zn and Cu, Zn(OAc)_2_·2H_2_O or Cu(OAc)_2_·H_2_O in methanol was dropwise added to **1a**, **b**, or **d** in ethanol under nitrogen gas. The reaction mixture was refluxed for 4 hours and a light yellow or dark green solid was formed. Complexes **2a**–**d** were obtained by filtration and washed with cold methanol. For reactions using Co salt, Co(OAc)_2_·4H_2_O and **1d**, **g**, or **e** was gradually added to methanol under nitrogen gas. The reaction mixture was stirred at 0 °C for 40 min and a brick-red precipitate was formed. Complexes **2e–g** were isolated by the similar purifying method as described above. The yield of Zn complex **2a** (81%) is slightly lower than the Cu complex **2b** (89%). Compounds **1b** and **1d** reacted with Cu to afford **2c** and **2d** in the yields of 83% and 94%, respectively. The reaction affinity between Co and selected salens was higher than Zn and Cu complexes, for instance, **2e** (96%), **2f** (98%), and **2g** (95%). A *tert*-butyl group played an important role as an electron-donating moiety for increasing the yield (**2d**–**g**). The slightly higher yield of **2f** over **2e** suggested a relatively more effective preparation for chiral salen complexes.

The HKR of epichlorohydrin with water was selected as a classical model to evaluate the catalytic activity of Co-unsymmetrical salen complexes **2e**, **2f**, and **2g** for the asymmetric ring opening of epoxides. Enantiomeric excess (ee) results of 3-chloro-1,2-propanediol from the HKR reactions were summarized in Table 2. The complex **2** (0.5 mmol) and trace amount of glacial acetic acid were added to dry dichloromethane. The mixture solution was evaporated after the reaction color changed from orange-red to dark brown in 30 minutes. Racemic epichlorohydrin and deionized water were subsequently added to the reaction and stirred for 18 hours at 0 °C. Upon the reaction completion, 3-chloro-1,2-propanediol in highly enantioenriched structure was afforded using chiral catalyst **2f**, while non-chiral catalysts **2e** and **2g** displayed nonenantioselective results (Table 2).

**Table 2:** HKR of epichlorohydrin with water catalyzed by **2**.^a^

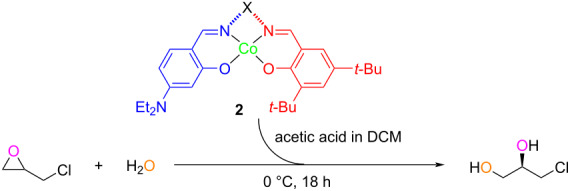		

Entry	Catalyst	ee (%)^b^

1	**2e**	0
2	**2f**	98
3	**2g**	0

^a^Reaction conditions: **2** (0.5 mmol, 0.5 mol % of deionized water), DCM (8 mL), acetic acid (5 mmol) were stirred for 30 min at rt, epichlorohydrin (167 mmol, 1.8 equiv) in deionized water (1.65 mL, 92 mmol, 1 equiv) was added to the reaction system at 0 °C and stirred for 18 h for completion; ^b^determined by chiral HPLC analysis, [α]_D_^23^ +22.30 (*c* 1, MeOH).

To broaden the use of our chiral catalyst, α-aryloxy alcohols were thereafter synthesized through the KR of epichlorohydrin with different phenols using chiral Co–salen catalyst **2f** (Table 3). *meta*-Substituted methylphenol showed less reactivity and selectivity (Table 3, entry 2), while *tert*-butyl monosubstitution at the *para*-position on the phenol slightly increased in light of the yield and ee (Table 3, entry 3). Bulky phenol afforded no product (**3e**), which is in good agreement with the suggested Co–salen catalytic mechanism [6]. Phenols with both electron-donating and electron-withdrawing moieties participated in the asymmetric ring opening of epichlorohydrin and provided α-aryloxy alcohols in an overall high yield and a complete enantioselectivity.

**Table 3:** Synthesis of α-aryloxy alcohols **3** by KR of epichlorohydrin with phenols catalyzed by complex **2f**.^a,b^.

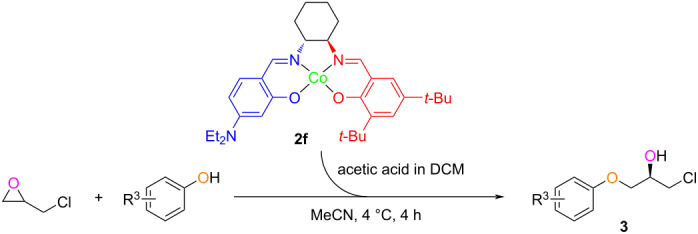				

Entry	ID	R^3^	Yield (%)^c^	ee (%)^d^

1	**3a**	H	60	98
2	**3b**	*m*-CH_3_	67	93
3	**3c**	*p*-*t*-Bu	75	99
4	**3d**	*p*-CHO	56	96
5	**3e**	di-*o*-*t*-Bu; *p*-CH_3_	0	–

^a^Reaction conditions: Complex **2f** (0.1 mmol, 0.5 mol % of phenol), DCM (2 mL), and acetic acid (1 mmol) were stirred for 30 min at rt, epichlorohydrin (44.4 mmol, 2.22 equiv) in MeCN (1.1 mL) was added to the reaction system at 4 °C and stirred for 20 min, followed by the addition of the phenol (20 mmol, 1 equiv) and stirring at 4 °C for 4 h for completion; ^b^see ref. [21] for method development; ^c^isolated yields based on alcohol; ^d^determined by chiral HPLC analysis.

## Conclusion

In summary, we mechanochemically synthesized unsymmetrical salens **1** for preparing metal–salen catalysts **2** for the first time. The use of grinding technology provided salens **1** in an overall higher yield in comparison to the self-made ball milling. Faster RPM (over 700 rev/min) might be necessary to increase the reaction efficiency through a ball milling technology. Chelating ability of **1** with different metals was explored and metal–salen complexes **2a**–**g** were highly yielded, demonstrating an intimate affinity of unsymmetrical salens chelating with metals. The HKR of epichlorohydrin with water catalyzed by Co–salens **2** was studied and chiral **2f** showed an outstanding catalytic ability to afford the diol product in high ee (98%). A library of α-aryloxy alcohols was thereafter synthesized through the asymmetric ring opening of epichlorohydrin with different phenols in the presence of **2f** (0.5 mol %), resulting in good yields and high ee (up to 99%). Further application of chiral Co–salen complexes and their reaction mechanism will be addressed in the due course.

## Supporting Information

File 1 Experimental section and copies of spectra.
