# Supplementary material for: Mechanochemical synthesis of unsymmetrical salens for the preparation of Co–salen complexes and their evaluation as catalysts for the synthesis of α-aryloxy alcohols via asymmetric phenolic kinetic resolution of terminal epoxides
Source: Beilstein J Org Chem. 2022 Oct 10;18:1416–23. doi: 10.3762/bjoc.18.147 (PMC9577384; doi:10.3762/bjoc.18.147)
Supplement: File 1 — Experimental section and copies of spectra. [file Beilstein_J_Org_Chem-18-1416-s001.pdf]

BEILSTEIN JOURNAL OF ORGANIC CHEMISTRY

Supporting Information

for

Mechanochemical synthesis of unsymmetrical salens for the preparation of Co–salen complexes and their evaluation as catalysts for the synthesis of α-aryloxy alcohols via asymmetric phenolic kinetic resolution of terminal epoxides

Shengli Zuo, Shuxiang Zheng, Jianjun Liu and Ang Zuo

Beilstein J. Org. Chem. **2022,** *18,* 1416–1423. doi:10.3762/bjoc.18.147

Experimental section and copies of spectra

License and Terms: This is a supporting information file under the terms of the Creative Commons Attribution License (https://creativecommons.org/licenses/by/4.0). Please note that the reuse, redistribution and reproduction in particular requires that the author(s) and source are credited and that individual graphics may be subject to special legal provisions.
The license is subject to the *Beilstein Journal of Organic Chemistry* terms and conditions: (https://www.beilstein-journals.org/bjoc/terms)

Table of contents

General experimental details……………………………………………….……………………S1

^{1}H and ^{13}C NMR spectra…………………………………………………….……………………S19

IR spectra……………………………………………………………………………………...S43

General experimental details

All starting materials were purchased from commercial sources and used without further treatment. *Trans*-cyclohexanediamine and ethylenediamine unilateral hydrochloride were synthesized according to the method reported in the literature [1-3]. Analytical thin-layer chromatography (TLC) was performed on GF254 silica gel. ^{1}H NMR spectra were obtained and recorded in a Bruker Avance III spectrometer at 400 MHz with tetramethylsilane as an internal standard. Multiplicities are indicated as s (singlet), d (doublet), t (triplet), q (quartet), dd (double doublet), m (multiplet), and b (broad). Coupling constants (*J*) are reported in hertz (Hz). General NMR data were obtained at 25 °C (298.15 K). Infrared (IR) spectra were measured using an AVATAR-370 as KBr pellets, (v [cm^{-1}]). High-resolution mass (HRMS) spectra were performed on a Waters Xevo G2 Qtof spectrometer. Optical rotation was determined on a WXG-4 polarimeter. Chiral HPLC analysis was carried out with a Chiracel OD column (24 cm × 0.46 mm, Chiral Technologies, Inc.) equipped with a Waters 510 pump (flow rate at 1 mL/min) and a UV detector.

The volume of the filling container of the self-made ball mill is 37 mL. The grinding media were 16.6 g. The working speed is 700r/min, and the maximum speed of the machine is 3500 r/min. XD-3420 permanent magnet DC motor DC: 12 V.

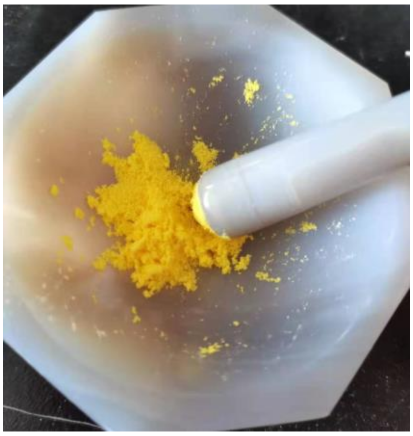

Figure S1: Grinding using mortar and pestle.

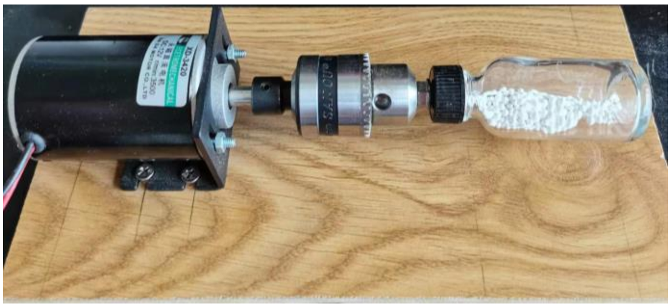

Figure S2: Self-made ball mill.

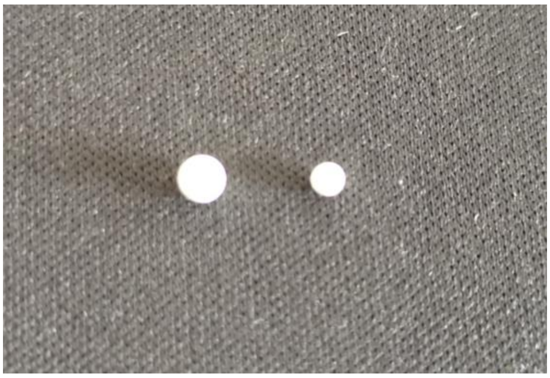

Figure S3: Zirconia and alumina balls.

General procedure for the synthesis of unsymmetrical salen ligands 1a–h

The synthesis of asymmetric salen-type ligands was performed using a one-pot two-step method. Firstly, salicylaldehyde (1 mmol) and *trans*-cyclohexanediamine or ethylenediamine unilateral salt (2 mmol), were grinded in an agate mortar for 10 min. In the second step, 2 equivalents of triethylamine (4 mmol), methanol (0.12 μL/mg), and another salicylaldehyde (1 mmol) were added to the reaction mixture, and grinded for further 20 min. A yellow sticky solid was obtained and the reaction mixture was dissolved in CH_2Cl_2. The crude product was concentrated to remove the solvent, and purified by flash column chromatography with different ratios of *n*-hexane/ethyl acetate as the eluent. Monitored by TLC, pure unsymmetrical salens **1[a]–h** were obtained.

In the self-made ball mill procedure, all materials were fed according to the above method. The reaction was milled for 1 hour in the first stage, and was milled for an addition hour after adding reagents in the second stage. Compounds **1a–h** were obtained by purification according to the above method.

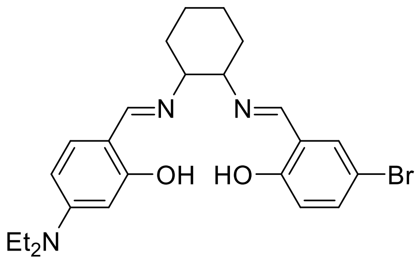

1a

Following the general procedure, flash column chromatography was performed using hexanes/ethyl acetate 2:1 as eluent. Compound **1a**

(4-bromo-2-((*E*)-((2-(((*E*)-4-(diethylamino)-2-hydroxybenzylidene)amino)cyclohexyl)imino)

methyl)phenol) was obtained as bright yellow oily liquid (0.895 g, 95% in yield): ^{1}H NMR (400 MHz, $CDCl_3$) δ: 13.20 (s, 1H), 8.20 (s, 1H), 7.73 (s, 1H), 7.35 – 7.27 (m, 2H), 6.90 – 6.83 (m, 1H), 6.80 – 6.73 (m, 1H), 6.48 – 6.31 (m, 1H), 6.15 – 6.08 (m, 1H), 3.37 (q, J = 6.2 Hz, 4H), 3.31 – 3.23 (m, 2H), 2.07 – 1.96 (m, 2H), 1.93 – 1.87 (m, 2H), 1.70 – 1.63 (m, 2H), 1.48 – 1.42 (m, 2H), 1.17 (t, J = 5.8 Hz, 6H). ^{13}C NMR (101 MHz, $CDCl_3$) δ: 165.05, 163.54, 163.02, 160.31, 151.42, 134.75, 133.66, 132.94, 120.14, 118.87, 109.97, 108.14, 103.12, 97.97, 73.02, 70.94, 44.51, 33.26, 33.11, 24.40, 24.19, 12.77. IR (KBr)/cm^{-1} v: 3416, 2970, 2930, 2856, 1614, 1522, 1450, 1377, 1344, 1292, 1240, 1180, 1130, 1078, 856, 785, 740,704. HRMS: calc for $C_{24}H_{30}BrN_3O_2$ ($[M + H]^+$) 472.1599; found 472.1590.

Compound **1a** was obtained as bright yellow oily liquid (0.773 g, 82% in yield) by ball milling.

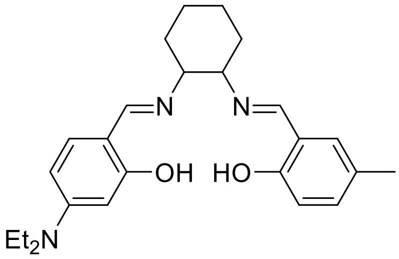

1b

Following the general procedure, flash column chromatography was performed using hexanes/ethyl acetate 9:5 as eluent. Compound **1b** (2-((*E*)-((2-(((*E*)-4-(diethylamino)-2-hydroxybenzylidene)amino)cyclohexyl)imino)methyl)-4-methylphenol) was obtained as bright yellow oily liquid (0.766 g, 94% in yield): ^{1}H NMR (400 MHz, $CDCl_3$) δ: 13.09 (s, 1H), 8.21 (s, 1H), 7.91 (s, 1H), 7.07 – 7.00 (m, 1H), 6.96 – 6.91 (m, 1H), 6.90 – 6.84 (m, 1H), 6.82 – 6.76 (m, 1H), 6.06 – 6.02 (m, 2H), 3.30 (q, J = 7.1

Hz, 4H), 3.25 – 3.17 (m, 2H), 2.20 (s, 3H), 1.97 – 1.87 (m, 3H), 1.86 – 1.78 (m, 2H), 1.71 – 1.61 (m, 2H), 1.48 – 1.38 (m, 2H), 1.13 (t, J = 7.0 Hz, 6H). ^{13}C NMR (101 MHz, $CDCl_3$) δ: 165.84, 164.84, 162.78, 158.86, 151.57, 133.15, 132.93, 131.74, 127.61, 118.50, 116.53, 108.23, 103.11, 98.11, 73.10, 70.73, 44.55, 33.35, 33.28, 24.50, 24.28, 20.37, 12.80. IR (KBr)/cm^{-1} ν: 3442, 2972, 2928, 2856, 1616, 1522, 1493, 1375, 1355, 1278, 1240, 1132, 1093, 939, 820, 781. HRMS: calc for $C_{25}H_{33}N_3O_2$ ([M + H]$^+$) 408.2651; found 408.2651.

Compound **1b** was obtained as bright yellow oily liquid (0.578 g, 71% in yield) by ball milling.

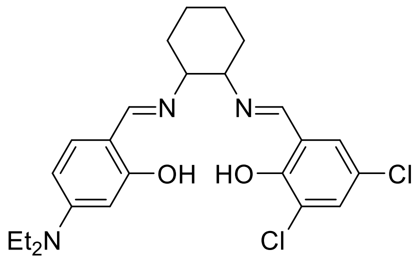

1c

Following the general procedure, flash column chromatography was performed using hexanes/ethyl acetate 2:1 as eluent. Compound **1c** (2,4-dichloro-6-((*E*)-((2-(((*E*)-4-(diethylamino)-2-hydroxybenzylidene)amino)cyclohexyl)imino)methyl)phenol) was obtained as light yellow solid (0.812 g, 88% in yield): Melting point: 64-66 °C. ^{1}H NMR (400 MHz, $CDCl_3$) δ: 8.15 (s, 1H), 7.90 (s, 1H), 7.37 – 7.31 (m, 1H), 7.05 – 7.01 (m, 1H), 6.92 – 6.86 (m, 1H), 6.16 – 6.06 (m, 2H), 3.34 (q, J = 14.0, 6.9 Hz, 5H), 3.23 – 3.14 (m, 1H), 2.00 – 1.93 (m, 2H), 1.90 – 1.84 (m, 2H), 1.74 – 1.62 (m, 2H), 1.49 – 1.41 (m, 2H), 1.15 (t, J = 7.0 Hz, 6H). ^{13}C NMR (101 MHz, $CDCl_3$) δ: 165.10, 163.65, 163.09, 157.77, 152.13, 133.63, 132.42, 129.45, 123.04, 122.26, 119.08, 103.66, 97.98, 86.30, 72.08, 70.60,

44.72, 33.23, 32.99, 24.39, 24.16, 12.81. IR (KBr)/cm^{-1} v: 3442, 2970, 2930, 2856, 2361, 1736, 1616, 1522, 1477, 1375, 1346, 1277, 1240, 1128, 1093, 820, 698, 627. HRMS: calc for $C_{24}H_{29}Cl_2N_3O_2$ ([M + H]$^+$) 462.1715; found 462.1715.

Compound **1c** was obtained as light yellow solid (0.711 g, 77% in yield) by ball milling.

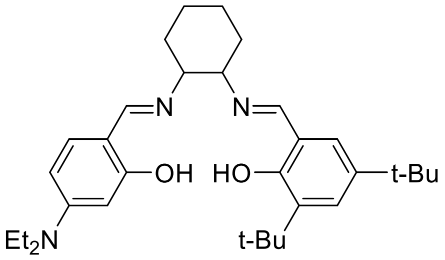

1d

Following the general procedure, flash column chromatography was performed using hexanes/ethyl acetate 5:2 as eluent. Compound **1d** (2,4-di-*tert*-butyl-6-((*E*)-((2-(((*E*)-4-(diethylamino)-2-hydroxybenzylidene)amino)cyclohexyl)imino)methyl)phenol) was obtained as bright yellow oily liquid (0.798 g, 79% in yield): ^{1}H NMR (400 MHz, $CDCl_3$) δ: 8.26 (s, 1H), 7.92 (s, 1H), 7.33 – 7.30 (m, 1H), 6.99 – 6.96 (m, 1H), 6.88 – 6.83 (m, 1H), 6.08 – 6.03 (m, 2H), 3.32 (q, J = 7.1 Hz, 4H), 3.27 – 3.18 (m, 2H), 2.11 – 1.61 (m, 8H), 1.44 (s, 9H), 1.26 (s, 9H), 1.14 (t, J = 7.1 Hz, 6H). ^{13}C NMR (101 MHz, $CDCl_3$) δ: 166.66, 166.14, 162.75, 158.11, 151.81, 139.98, 136.37, 133.31, 126.87, 126.30, 117.96, 108.24, 103.17, 98.25, 72.87, 70.38, 44.59, 35.08, 34.18, 33.55, 33.11, 31.59, 29.59, 24.58, 24.37, 12.83. IR (KBr)/cm^{-1} v: 3408, 2955, 2930, 2860, 2361, 1618, 1522, 1470, 1441, 1377, 1358, 1240, 1130, 1094, 827, 785, 706, 644. HRMS: calc for $C_{32}H_{47}N_3O_2$ ([M + H]$^+$) 506.3746; found 506.3742.

Compound **1d** was obtained as bright yellow oily liquid (0.667 g, 66% in yield) by ball milling.

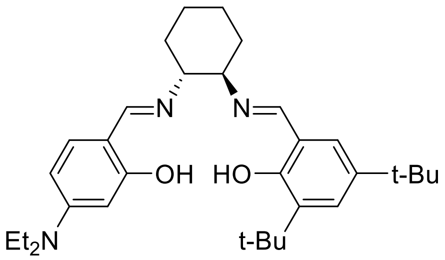

1e

Following the general procedure, flash column chromatography was performed using hexanes/ethyl acetate 5:2 as eluent. Compound **1e** (2,4-di-*tert*-butyl-6-((*E*)-(((1*R*,2*R*)-2-(((*E*)-4-(diethylamino)-2-hydroxybenzylidene)amino)cyclohexyl)imino)methyl)phenol) was obtained as bright yellow oily liquid (0.819 g, 81% in yield): ^{1}H NMR (400 MHz, $CDCl_3$) δ: 13.67 (s, 2H), 8.25 (s, 1H), 7.93 (s, 1H), 7.33 – 7.30 (m, 1H), 6.99 – 6.95 (m, 1H), 6.88 – 6.83 (m, 1H), 6.08 – 6.02 (m, 2H), 3.32 (q, *J* = 7.0 Hz, 4H), 3.27 – 3.17 (m, 2H), 2.10 – 1.55 (m, 8H), 1.43 (s, 9H), 1.25 (s, 9H), 1.14 (t, *J* = 7.0 Hz, 6H). ^{13}C NMR (101 MHz, $CDCl_3$) δ: 166.78, 166.13, 162.79, 158.14, 151.76, 139.99, 136.39, 133.25, 126.88, 126.31, 117.98, 108.35, 103.14, 98.30, 72.94, 70.43, 44.60, 35.10, 34.20, 33.56, 33.17, 31.61, 29.61, 24.60, 24.40, 12.85. IR (KBr)/cm^{-1} ν: 2958, 2864, 1622, 1522, 1469, 1442, 1360, 1242, 1130, 1095, 827, 785, 773, 706, 644, 445. HRMS: calc for $C_{32}H_{47}N_3O_2$ ($[M + H]^+$) 506.3746; found 506.3745.

Compound **1e** was obtained as bright yellow oily liquid (0.687 g, 68% in yield) by ball milling.

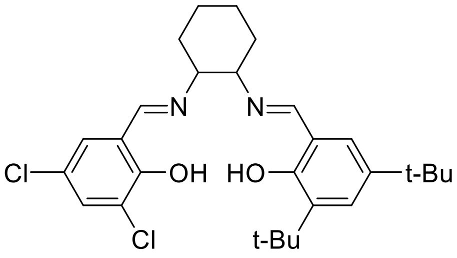

1f

Following the general procedure, flash column chromatography was performed using hexanes/ethyl acetate 15:1 as eluent. Compound **1f** (2,4-di-*tert*-butyl-6-((*E*)-((2-(((*E*)-3,5-dichloro-2-hydroxybenzylidene)amino)cyclohexyl)imino)methyl)phenol) was obtained as bright yellow oily liquid (0.723 g, 72% in yield): ^{1}H NMR (400 MHz, $CDCl_3$) δ: 13.48 (s, 1H), 8.26 (s, 1H), 8.18 (s, 1H), 7.37 – 7.34 (m, 1H), 7.34 – 7.31 (m, 1H), 7.03 – 7.00 (m, 1H), 7.00 – 6.98 (m, 1H), 3.46 – 3.38 (m, 1H), 3.30 – 3.22 (m, 1H), 2.07 – 1.58 (m, 8H), 1.44 (s, 9H), 1.26 (s, 9H). ^{13}C NMR (101 MHz, $CDCl_3$) δ: 166.39, 163.51, 157.93, 157.50, 140.36, 136.57, 132.29, 129.28, 127.32, 126.27, 122.94, 122.33, 119.20, 117.72, 72.39, 72.09, 35.11, 34.22, 33.32, 32.76, 31.56, 29.53, 24.26. IR (KBr)/cm^{-1} ν: 3417, 2953, 2930, 2860, 2361, 1737, 1630, 1452, 1375, 1362, 1178, 1097, 1043, 866, 741. HRMS: calc for $C_{28}H_{36}Cl_2N_2O_2$ ([M + H]$^+$) 503.2232; found 503.2231.

Compound **1f** was obtained as bright yellow oily liquid (0.573 g, 57% in yield) by ball milling.

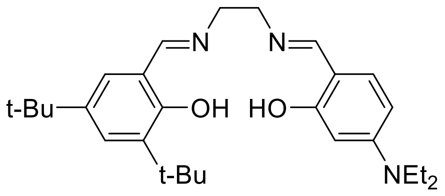

1g

Following the general procedure, flash column chromatography was performed using hexanes/ethyl acetate 1:3 as eluent. Compound **1g** (2,4-di-*tert*-butyl-6-((*E*)-((2-(((*E*)-4-(diethylamino)-2-hydroxybenzylidene)amino)ethyl)imino)methyl)phenol) was obtained as light yellow solid (0.731 g, 81% in yield): Melting point: 142-144 ^{o}C. ^{1}H NMR (400 MHz, $CDCl_3$) δ: 13.60 (s, 1H), 8.35 (s, 1H), 8.02 (s, 1H), 7.38 – 7.34 (m, 1H), 7.07 – 7.03 (m, 1H), 6.99 – 6.94 (m, 1H), 6.17 – 6.10 (m, 2H), 3.88 – 3.80 (m, 4H), 3.35 (q, 4H), 1.44 (s, 9H), 1.28 (s, 9H), 1.17 (t, J = 7.1 Hz, 6H). ^{13}C NMR (101 MHz, $CDCl_3$) δ: 167.75, 164.49, 158.17, 151.83, 140.16, 136.67, 133.31, 128.66, 127.17, 126.27, 117.91, 108.27, 103.34, 98.26, 60.22, 59.76, 57.86, 44.67, 35.16, 34.27, 31.64, 29.58, 22.87, 12.86. IR (KBr)/cm^{-1} ν: 3449, 2968, 1616, 1520, 1350, 1130, 1076, 1040, 824, 787, 704. HRMS: calc for $C_{28}H_{41}N_3O_2$ ($[M + H]^+$) 452.3277; found 452.3278.

Compound **1g** was obtained as light yellow solid (0.650 g, 72% in yield) by ball milling.

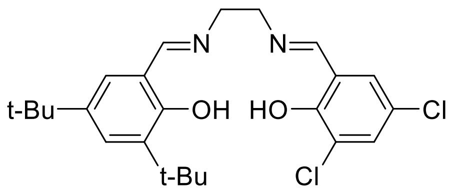

1h

Following the general procedure, flash column chromatography was performed using hexanes/ethyl acetate 5:1 as eluent. Compound **1h** (2,4-di-*tert*-butyl-6-((*E*)-((2-(((*E*)-3,5-dichloro-2-hydroxybenzylidene)amino)ethyl)imino)methyl)phenol) was obtained as bright yellow oily liquid (0.681 g, 76% in yield): ^{1}H NMR (400 MHz, $CDCl_3$) δ: 14.23 (s, 1H), 13.41 (s, 1H), 8.35 (s, 1H), 8.26 (s, 1H), 7.40 – 7.36 (m, 2H), 7.13 – 7.10 (m, 1H), 7.07 – 7.04 (m, 1H), 4.01 – 3.97 (m, 2H), 3.95 – 3.90 (m, 2H), 1.42 (s, 9H), 1.28 (s, 9H). ^{13}C NMR (101 MHz, $CDCl_3$) δ: 168.15, 165.28, 158.08, 156.93, 140.49, 136.87, 132.49, 129.36, 127.54, 126.33, 122.97, 122.81, 119.53, 117.81, 59.31, 59.20, 35.20, 34.31, 31.64, 29.57. IR (KBr)/cm^{-1} ν: 3419, 2962, 2869, 1631, 1466, 1441, 1361, 1271, 1213, 1174, 1041, 839, 731. HRMS: calc for $C_{24}H_{30}Cl_2N_2O_2$ ($[M + H]^+$) 449.1762; found 449.1760.

Compound **1h** was obtained as bright yellow oily liquid (0.547 g, 61% in yield) by ball milling.

General procedure for the synthesis of metal–salen complexes 2a–g

A. A round-bottomed flask was charged with ligand **1a**, **b** or **d** (1 mmol) in EtOH (7 mL) and $Zn(OAc)_2{\cdot}2H_2O$ or $Cu(OAc)_2{\cdot}H_2O$ (1 mmol) in methanol (12 mL) were added dropwise via a constant pressure dropping funnel to the ligand solution over 1 minute. After refluxing for 4 h, a light yellow or dark green solid was produced. The reaction mixture was then filtered and washed with cold methanol (2 × 20 mL) to afford complexes **2a–d**.

B. Ligand **1d**, **g** or **e** (1 mmol) and $Co(OAc)_2 \cdot 4H_2O$ (0.3 g, 1.2 mmol) were placed in a 50 mL round-bottomed flask [4]. After purging the system with nitrogen, methanol (10 mL) was added as a solvent. The reaction mixture was stirred at 0 °C for 40 min, and a brick-red precipitate formed. The reaction mixture was then filtered and washed with cold methanol (2 × 20 mL) to afford complexes **2e–g**.

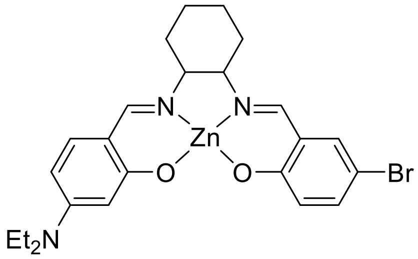

2a

Following the general procedure **A**, $Zn(OAc)_2 \cdot 2H_2O$ (0.22 g, 1 mmol) in 12 mL methanol. Compound **2a** was obtained as light yellow solid (0.468 g, 81% in yield): Melting point: 294-295 °C. IR (KBr)/cm^{-1} v: 3422, 2932, 1637, 1528, 1460, 1383, 1354, 1313, 1173, 822, 711, 638. HRMS: calc for $C_{24}H_{28}BrN_3O_2Zn$ ($[M + H]^+$) 534.0734; found 534.0739.

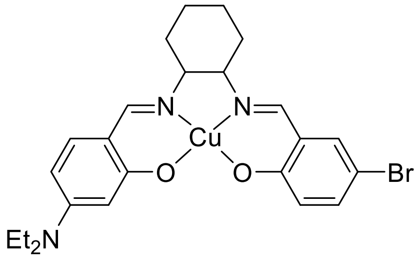

2b

Following the general procedure **A**, $Cu(OAc)_2 \cdot H_2O$ (0.2 g, 1 mmol) in 12 mL methanol. Compound **2b** was obtained as dark green solid (0.514 g, 89% in yield): Melting point: 240-241 °C. IR (KBr)/cm^{-1} v: 3423, 2934, 2361, 1601, 1516, 1460, 1381, 1348, 1315, 1246,

1173, 1138, 827, 714, 646. HRMS: calc for $C_{24}H_{28}BrCuN_3O_2$ ([M + H]$^+$) 533.0739; found 533.0737.

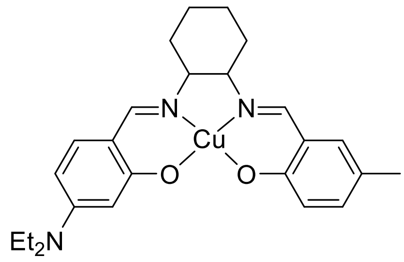

2c

Following the general procedure **A**, $Cu(OAc)_2{\cdot}H_2O$ (0.2 g, 1 mmol) in 12 mL methanol. Compound **2c** was obtained as dark green solid (0.426 g, 83% in yield): Melting point: 220-221 ºC. IR (KBr)/cm^{-1} *ν*: 3421, 2931, 2860, 1602, 1514, 1471, 1348, 1319, 1248, 1136, 825. HRMS: calc for $C_{25}H_{31}CuN_3O_2$ ([M + H]$^+$) 469.1790; found 469.1790.

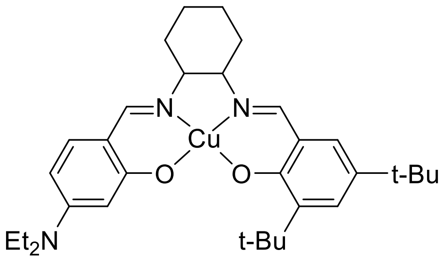

2d

Following the general procedure **A**, $Cu(OAc)_2{\cdot}H_2O$ (0.2 g, 1 mmol) in 12 mL methanol. Compound **2d** was obtained as dark green solid (0.575 g, 94% in yield): Melting point: 214-215 ºC. IR (KBr)/cm^{-1} *ν*: 2956, 1628, 1603, 1514, 1350, 1319, 1248, 1167, 1138, 825, 777. HRMS: calc for $C_{32}H_{45}CuN_3O_2$ ([M + H]$^+$) 567.2886; found 567.2885.

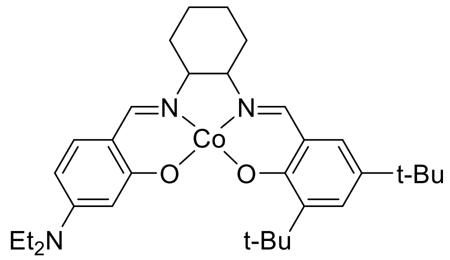

2e

Following the general procedure **B**. Compound **2e** was obtained as brick red solid (0.538 g, 96% in yield): Melting point: 236-238 °C. IR (KBr)/cm^{-1} *v*: 2952, 2865, 1595, 1509, 1356, 1318, 1252, 1172, 1138, 825, 785. HRMS: calc for $C_{32}H_{45}CoN_3O_2$ ($[M + H]^+$) 563.2922; found 563.2927.

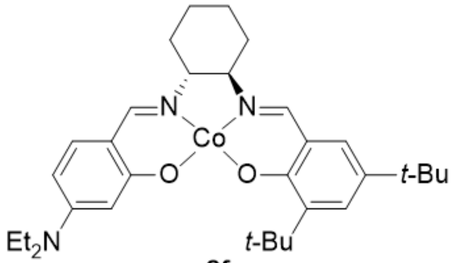

2f

Following the general procedure **B**. Compound **2f** was obtained as brick red solid (0.595 g, 98% in yield): Melting point: 234 °C. IR (KBr)/cm^{-1} *v*: 2946, 2865, 1598, 1510, 1353, 1319, 1253, 1170, 1138, 824, 780. HRMS: calc for $C_{32}H_{45}CoN_3O_2$ ($[M + H]^+$) 563.2922; found 563.2927.

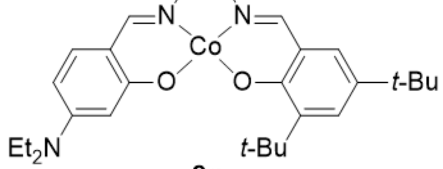

2g

Following the general procedure **B**. Compound **2g** was obtained as brick red solid (0.526 g,

95% in yield): Melting point: 212-214 °C. IR (KBr)/cm^{-1} ν: 2959, 2903, 2866, 1594, 1510, 1352, 1240, 1170, 1135, 1092, 945, 818, 773, 703. HRMS: calc for $C_{28}H_{39}CoN_3O_2$ ($[M + H]^+$) 509.2452; found 509.2454.

General procedure for HKR of epichlorohydrin catalyzed by salen complexes

Catalyst **2e**, **f** or **g** (0.5 mmol) was placed in a 100 mL round-bottomed flask and CH_2Cl_2 (8 mL) was added as solvent. Then, glacial acetic acid (290 μL, 5 mmol) was added and the mixture stirred for 30 min at room temperature. The color of the solution changed from orange-red to dark brown. The solution was concentrated to dryness under vacuum, and the crude Co(III)(OAc)–salen complex was obtained as a brown solid, which can be used without further purification [5]. Racemic epichlorohydrin (15.45 g, 167 mmol) and deionized water (1.65 mL) were added and the mixture stirred at 0 °C (ice–water bath) for 18 h. After completion of the reaction, atmospheric distillation was carried out. The fraction at 86 °C is an azeotrope of epichlorohydrin and water. The fraction at 116 °C was collected as the target substance and dried with anhydrous $MgSO_4$.

The target product catalyzed by **2f** was found to have an ee of 98% by chiral HPLC analysis (Chiralcel OD, 5% IPA in hexanes, 220 nm, 1 mL/min, t_R (major) = 5.7 min; t_R (minor) = 7.4 min). $[\alpha]_D^{23}$ +22.30 (*c* 1, MeOH).

General procedure for the synthesis of α-aryloxy alcohols 3a–e

The catalyst **2f** (0.0607 g, 0.1 mmol) was activated to Co(III)(OAc)–salen by the above procedure in a 50 mL flask. Epichlorohydrin (4.11 g, 44.4 mmol) and CH_3CN (1.1 mL) were

added to the activated Co(III)(OAc)–salen and the mixture was stirred until the solids have dissolved. Immerse the packed round-bottomed flask in a water bath at 4 °C for 20 min. Phenols with different substituents (1 equiv) were added to the system to start the reaction. The reaction mixture was stirred in an ice–water bath at 4 °C for 4 h and concentrated to remove the solvent. The crude product was loaded and purified by flash column chromatography with different ratios of *n*-hexane/ethyl acetate as eluent. Monitored by TLC, pure products **3a–d** were isolated.

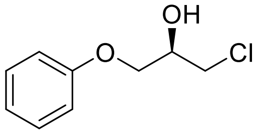

3a

Following the general procedure with phenol as raw material, flash column chromatography was performed using hexanes/ethyl acetate 4:1 as eluent. Compound **3a** ((*R*)-1-chloro-3-phenoxypropan-2-ol) was obtained. Recovered 2.23 g (60%) as yellowish oily liquid in 98% ee, determined by chiral HPLC analysis (Chiralcel OD, 7.5% IPA in hexanes, 210 nm, 1 mL/min, t_R (major) = 8.2 min; t_R (minor) = 12.8 min). ^{1}H NMR (400 MHz, $CDCl_3$) δ: 7.35 – 7.28 (m, 2H), 7.01 (t, *J* = 7.4 Hz, 1H), 6.94 (d, *J* = 7.9 Hz, 2H), 4.27 – 4.19 (m, 1H), 4.13 – 4.05 (m, 2H), 3.82 – 3.70 (m, 2H), 3.08 (br, 1H). ^{13}C NMR (101 MHz, $CDCl_3$) δ: 158.28, 129.68, 121.51, 114.66, 69.96, 68.58, 46.02. IR (KBr)/cm^{-1} ν: 3417, 3063, 3041, 2933, 2879, 1599, 1495, 1457, 1292, 1244, 1173, 1079, 1045, 813, 755, 692, 510. $[\alpha]^{23}_{D}$ -0.40 (589 nm, c=1, MeOH).

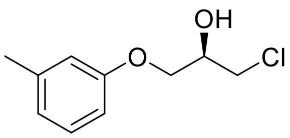

3b

Following the general procedure with *m*-cresol as raw material, flash column chromatography was performed using hexanes/ethyl acetate (4/1) as eluent. Compound **3b** ((*R*)-1-chloro-3-(*m*-tolyloxy)propan-2-ol) was obtained. Recovered 2.68 g (67%) as yellowish oily liquid in 93% ee, determined by chiral HPLC analysis (Chiralcel OD, 5% IPA in hexanes, 210 nm, 1 mL/min, t_R (major) = 10.7 min; t_R (minor) = 15.6 min). ^{1}H NMR (400 MHz, $CDCl_3$) δ: 7.19 (t, *J* = 7.8 Hz, 1H), 6.82 (d, *J* = 7.5 Hz, 1H), 6.74 (dd, *J* = 11.4, 3.2 Hz, 2H), 4.25 – 4.17 (m, 1H), 4.11 – 4.04 (m, 2H), 3.82 – 3.69 (m, 2H), 2.75 (br, 1H), 2.35 (s, 3H). ^{13}C NMR (101 MHz, $CDCl_3$) δ: 158.36, 139.84, 129.46, 122.41, 115.55, 111.58, 70.05, 68.55, 46.09, 21.62. IR (KBr)/cm^{-1} v: 3383, 3038, 2924, 2874, 1587, 1489, 1456, 1289, 1260, 1048, 929, 854, 776, 690. $[\alpha]^{23}{}_D$ -1.50 (589 nm, c=1, MeOH).

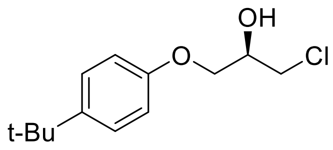

3c

Following the general procedure with 4-*tert*-butylphenol as raw material, flash column chromatography was performed using hexanes/ethyl acetate (8/1) as eluent. Compound **3c** ((*R*)-1-(4-(*tert*-butyl)phenoxy)-3-chloropropan-2-ol) was obtained. Recovered 3.63 g (75%) as yellowish oily liquid in 99% ee, determined by chiral HPLC analysis (Chiralcel OD, 10% IPA in hexanes, 220 nm, 1 mL/min, t_R (major) = 6.8 min; t_R (minor) = 10.4 min). ^{1}H NMR (400 MHz, $CDCl_3$) δ: 7.32 (d, *J* = 8.8 Hz, 2H), 6.86 (d, *J* = 8.8 Hz, 2H), 4.24 – 4.18 (m, 1H),

4.12 – 4.04 (m, 2H), 3.82 – 3.70 (m, 2H), 2.58 (br, 1H), 1.31 (s, 9H). ^{13}C NMR (101 MHz, $CDCl_3$) δ: 156.11, 144.41, 126.55, 114.22, 70.10, 68.65, 46.15, 34.29, 31.67. IR (KBr)/cm^{-1} ν: 3418, 2962, 2870, 1609, 1514, 1462, 1364, 1294, 1247, 1185, 1114, 1043, 829, 553. $[\alpha]^{23}{}_{D}$ +1.30 (589 nm, c=1, MeOH).

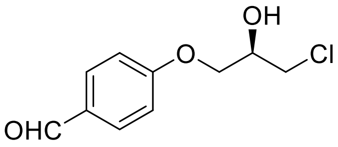

3d

Following the general procedure with *p*-hydroxybenzaldehyde as raw material, flash column chromatography was performed using hexanes/ethyl acetate (3/2) as eluent. Compound **3d** ((R)-4-(3-chloro-2-hydroxypropoxy)benzaldehyde) was obtained. Recovered 2.40 g (56%) as yellowish oily liquid in 96% ee, determined by chiral HPLC analysis (Chiralcel OD, 7.5% IPA in hexanes, 254 nm, 1 mL/min, t_R (major) = 12.6 min; t_R (minor) = 18.1 min). ^{1}H NMR (400 MHz, DMSO-*d6*) δ: 9.87 (s, 1H), 7.87 (d, *J* = 8.7 Hz, 2H), 7.16 (d, *J* = 8.7 Hz, 2H), 5.56 (d, *J* = 4.2 Hz, 1H), 4.27 – 4.21 (m, 2H), 4.21 – 4.13 (m, 2H), 3.35 (br, 1H). ^{13}C NMR (101 MHz, DMSO-*d6*) δ: 191.29, 163.48, 131.80, 129.73, 115.02, 69.55, 67.14. IR (KBr)/cm^{-1} ν: 3425, 2956, 2837, 2748, 1682, 1601, 1510, 1429, 1311, 1259, 1162, 1035, 836, 749, 652, 619, 515. $[\alpha]^{23}{}_{D}$ +5.35 (589 nm, c=1, MeOH).

References

1. Campbell, E. J.; Nguyen, S. T. *Tetrahedron Lett.* **2001**, *42*, 1221–1225.
2. Belokon, Y. N.; Maleev, V. I.; North, M.; Larionov, V. A.; TSavel'yeva, Y. F.; Nijland, A.; Nelyubina, Y. V. *ACS Catalysis* **2013**, *3*, 1951–1955.

3. Renehan, M. F.; Schanz, H.-J.; McGarrigle, E. M.; Dalton, C. T.; Daly, A. M.; Gilheany, D. G. *J. Mol. Catal. A Chem.* **2005**, *231*, 205–220.

4. Zheng, X.; Jones, C. W. Weck, M. *J. Am. Chem. Soc*. **2007**, *129*, 1105–1112.

5. Kim, S. K.; Jacobsen, E. N. *Angew. Chem. Int. Ed*. **2004**, *43,* 3952–3954.

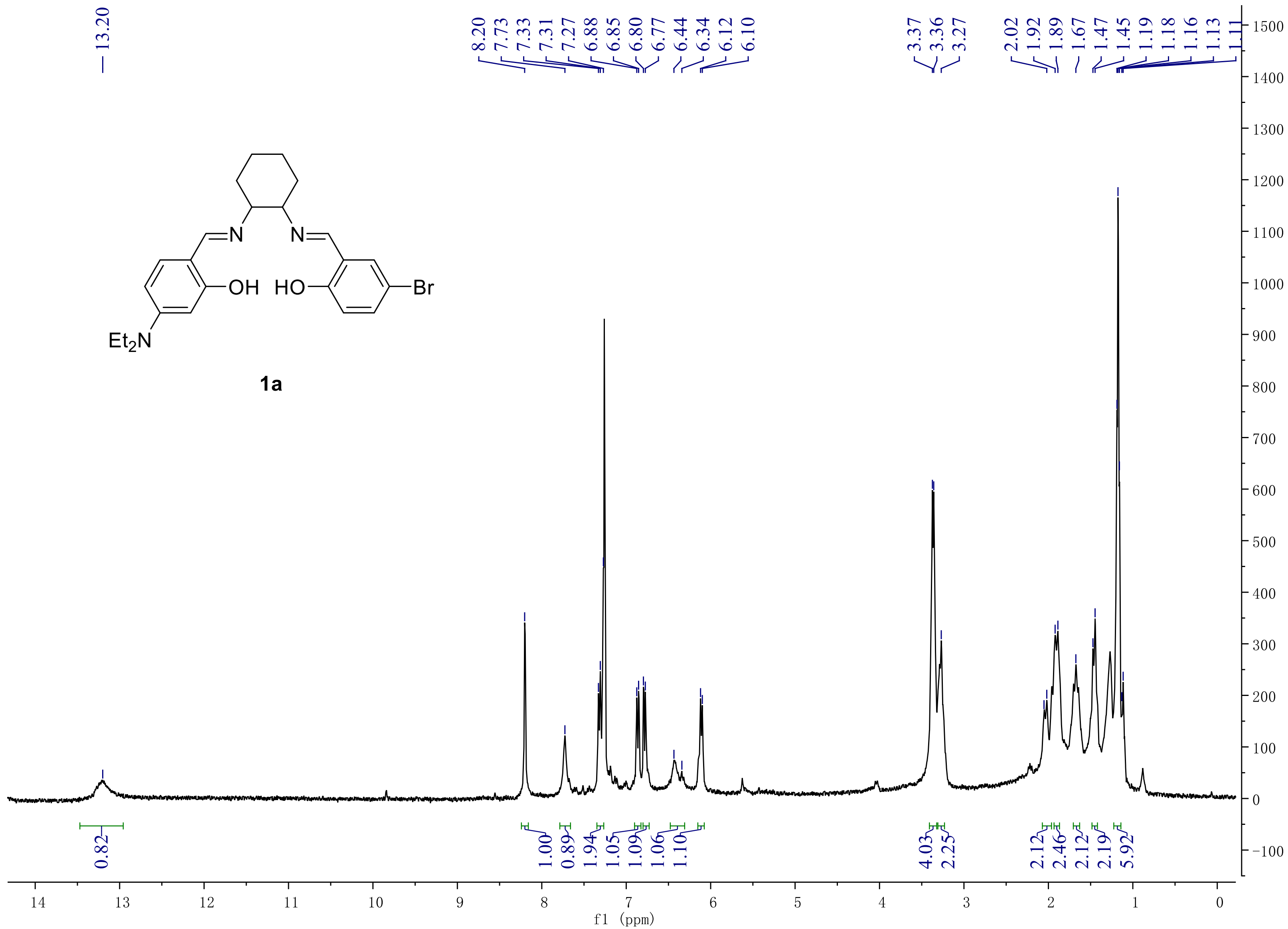

13.20
8.20
7.73
7.33
7.31
7.27
6.88
6.85
6.80
6.77
6.44
6.34
6.12
6.10
3.37
3.36
3.27
2.02
1.92
1.89
1.67
1.47
1.45
1.19
1.18
1.16
1.13
1.11
OH
HO
N
N
Br
Et_2N
1a
0.82
1.00
0.89
1.94
1.05
1.09
1.06
1.10
4.03
2.25
2.12
2.46
2.12
2.19
5.92
f1 (ppm)

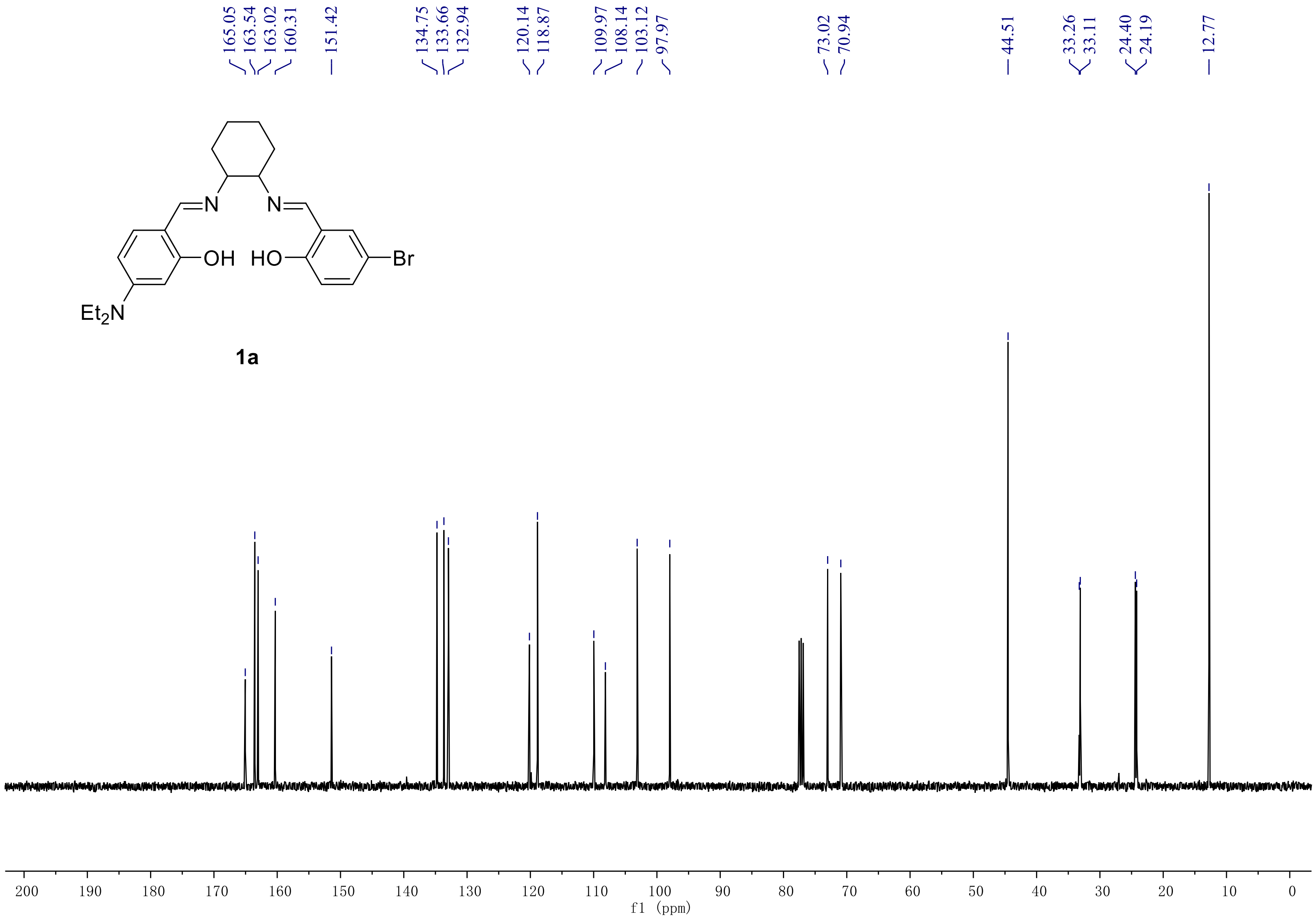
165.05
163.54
163.02
160.31
151.42
134.75
133.66
132.94
120.14
118.87
109.97
108.14
103.12
97.97
73.02
70.94
44.51
33.26
33.11
24.40
24.19
12.77
OH
HO
Br
Et_2N
N
N
1a
200 190 180 170 160 150 140 130 120 110 100 90 80 70 60 50 40 30 20 10 0
f1 (ppm)

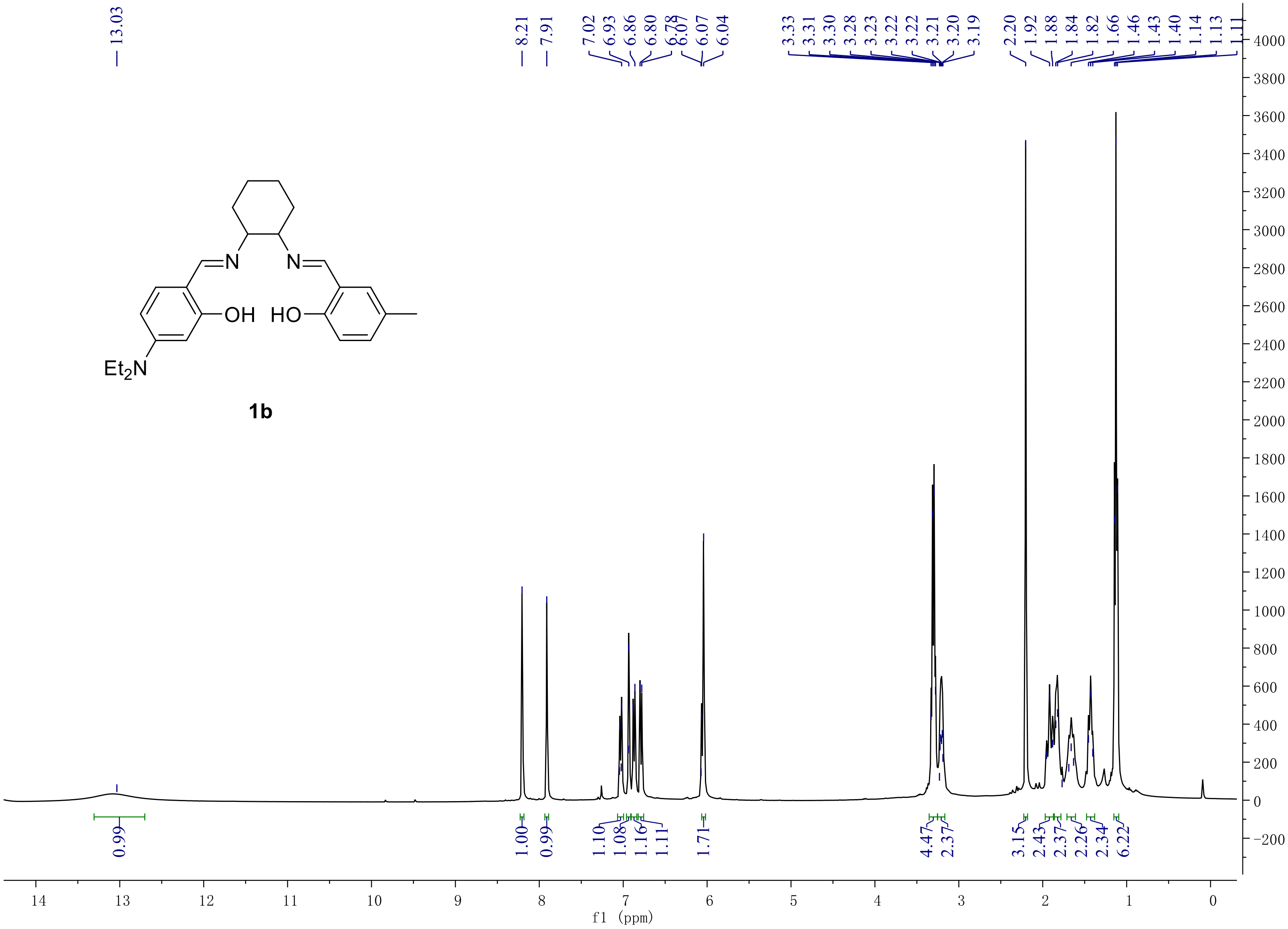

13.03
8.21
7.91
7.02
6.93
6.86
6.80
6.78
6.07
6.07
6.04
3.33
3.31
3.30
3.28
3.23
3.22
3.22
3.21
3.20
3.19
2.20
1.92
1.88
1.84
1.82
1.66
1.46
1.43
1.40
1.14
1.13
1.11
OH
HO
N
N
Et2N
1b
0.99
1.00
0.99
1.10
1.08
1.16
1.11
1.71
4.47
2.37
3.15
2.43
2.37
2.26
2.34
6.22
f1 (ppm)

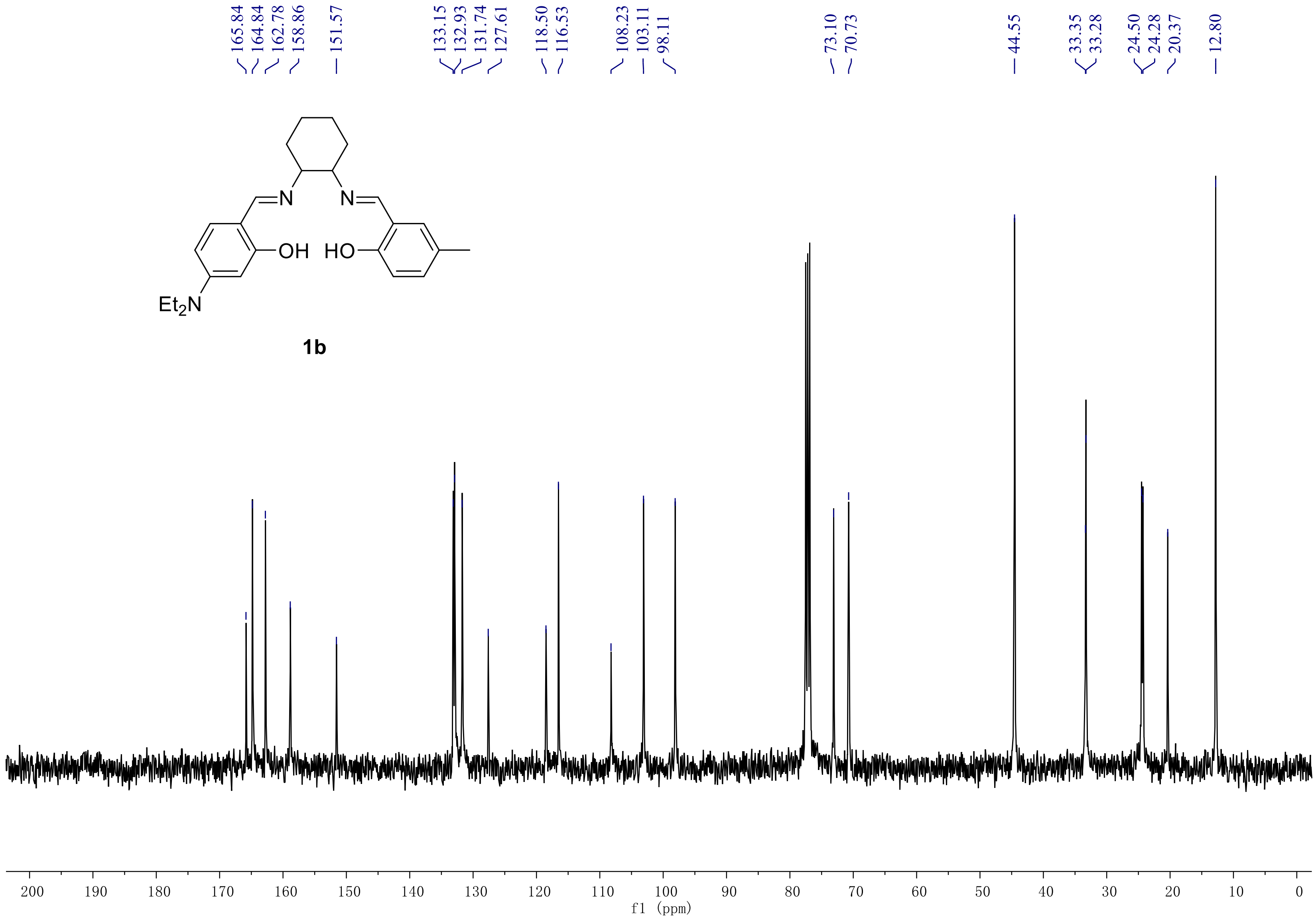
165.84
164.84
162.78
158.86
151.57
133.15
132.93
131.74
127.61
118.50
116.53
108.23
103.11
98.11
73.10
70.73
44.55
33.35
33.28
24.50
24.28
20.37
12.80
N
N
OH
HO
Et_2N
1b
200 190 180 170 160 150 140 130 120 110 100 90 80 70 60 50 40 30 20 10 0
f1 (ppm)

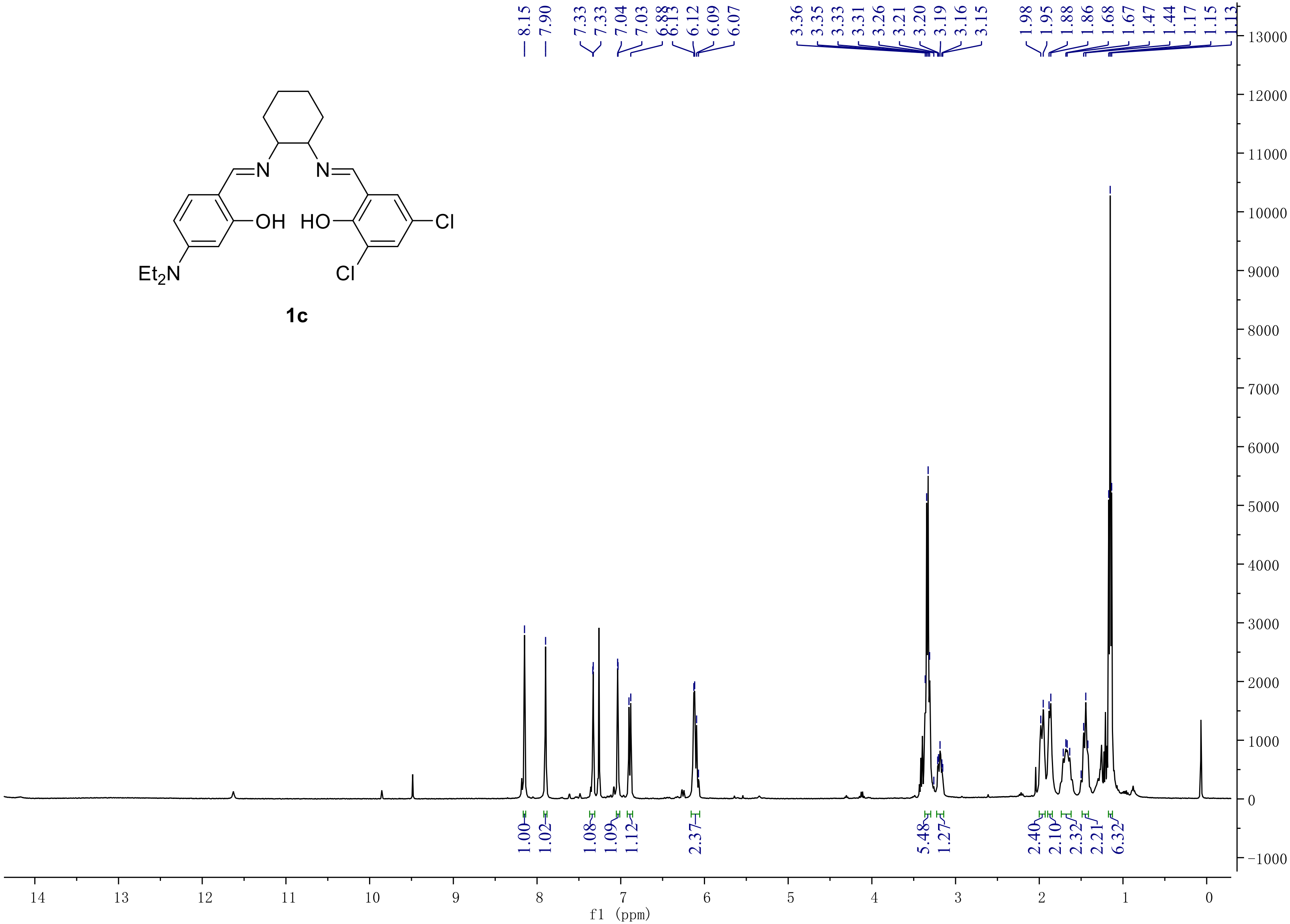
N
N
OH
HO
Cl
Cl
Et$_2$N
1c
8.15
7.90
7.33
7.33
7.04
7.03
6.88
6.13
6.12
6.09
6.07
3.36
3.35
3.33
3.31
3.26
3.21
3.20
3.19
3.16
3.15
1.98
1.95
1.88
1.86
1.68
1.67
1.47
1.44
1.17
1.15
1.13
1.00
1.02
1.08
1.09
1.12
2.37
5.48
1.27
2.40
2.10
2.32
2.21
6.32
f1 (ppm)
14
13
12
11
10
9
8
7
6
5
4
3
2
1
0
13000
12000
11000
10000
9000
8000
7000
6000
5000
4000
3000
2000
1000
0
−1000

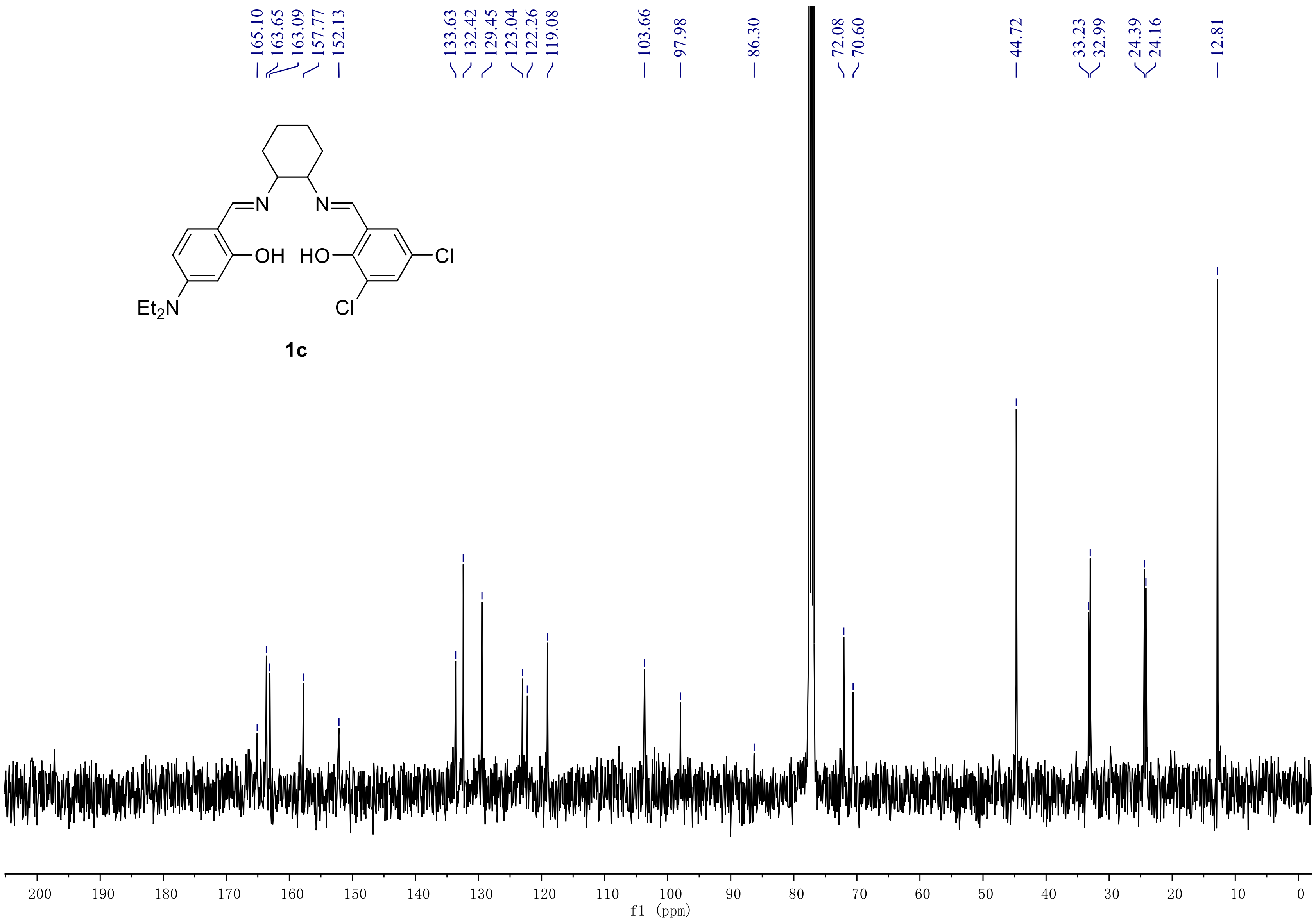
165.10
163.65
163.09
157.77
152.13
133.63
132.42
129.45
123.04
122.26
119.08
103.66
97.98
86.30
72.08
70.60
44.72
33.23
32.99
24.39
24.16
12.81
OH
HO
Cl
Cl
Et2N
N
N
1c
200
190
180
170
160
150
140
130
120
110
100
90
80
70
60
50
40
30
20
10
0
f1 (ppm)

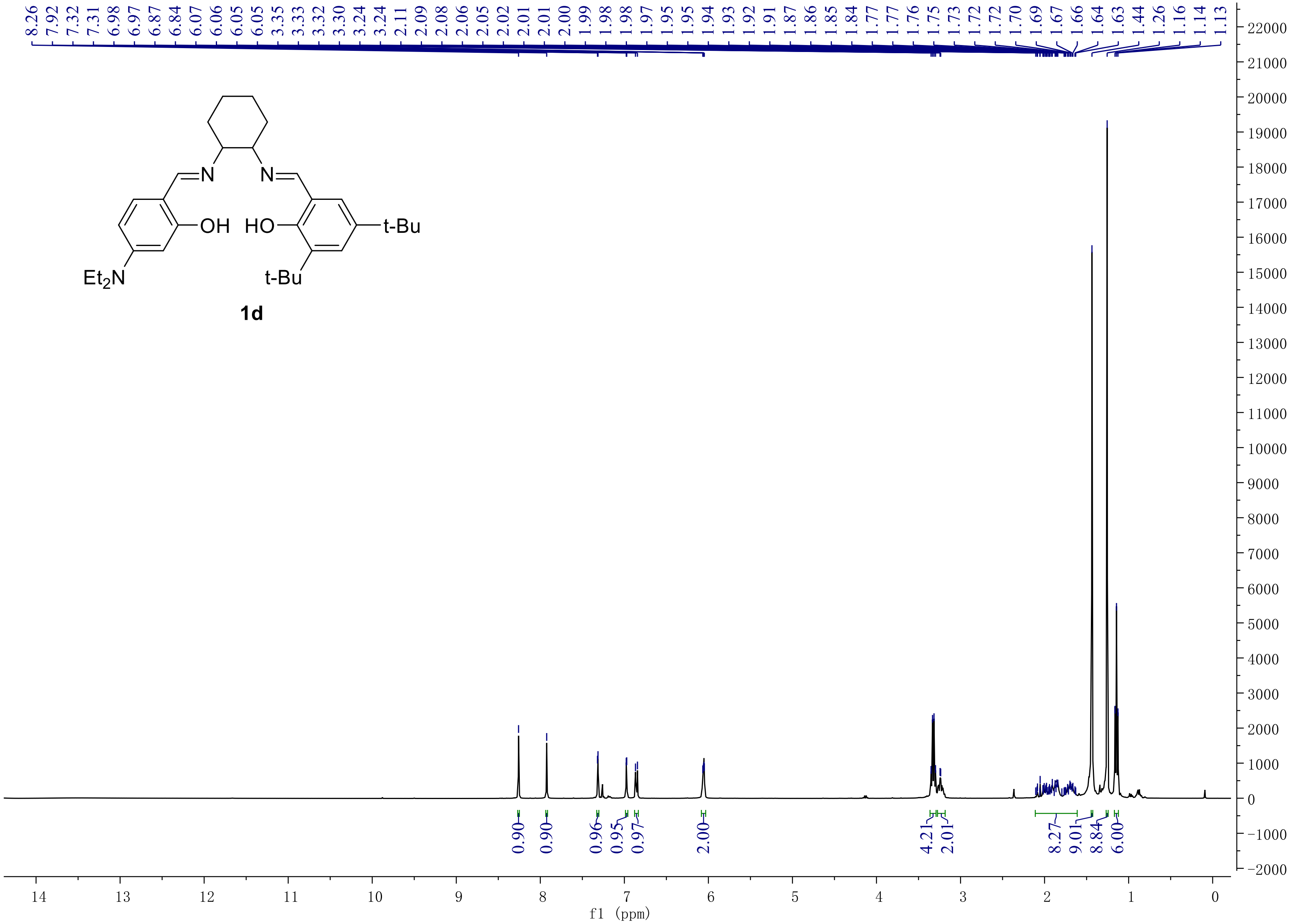
8.26 7.92 7.32 7.31 6.98 6.97 6.87 6.84 6.07 6.06 6.05 6.05 3.35 3.33 3.32 3.30 3.24 3.24 2.11 2.09 2.08 2.06 2.05 2.02 2.01 2.01 2.00 1.99 1.98 1.98 1.97 1.95 1.95 1.94 1.93 1.92 1.91 1.87 1.86 1.85 1.84 1.77 1.77 1.76 1.75 1.73 1.72 1.72 1.70 1.69 1.67 1.66 1.64 1.63 1.44 1.26 1.16 1.14 1.13
OH
HO
t-Bu
Et2N
t-Bu
1d
0.90 0.90 0.96 0.95 0.97 2.00 4.21 2.01 8.27 9.01 8.84 6.00
f1 (ppm)

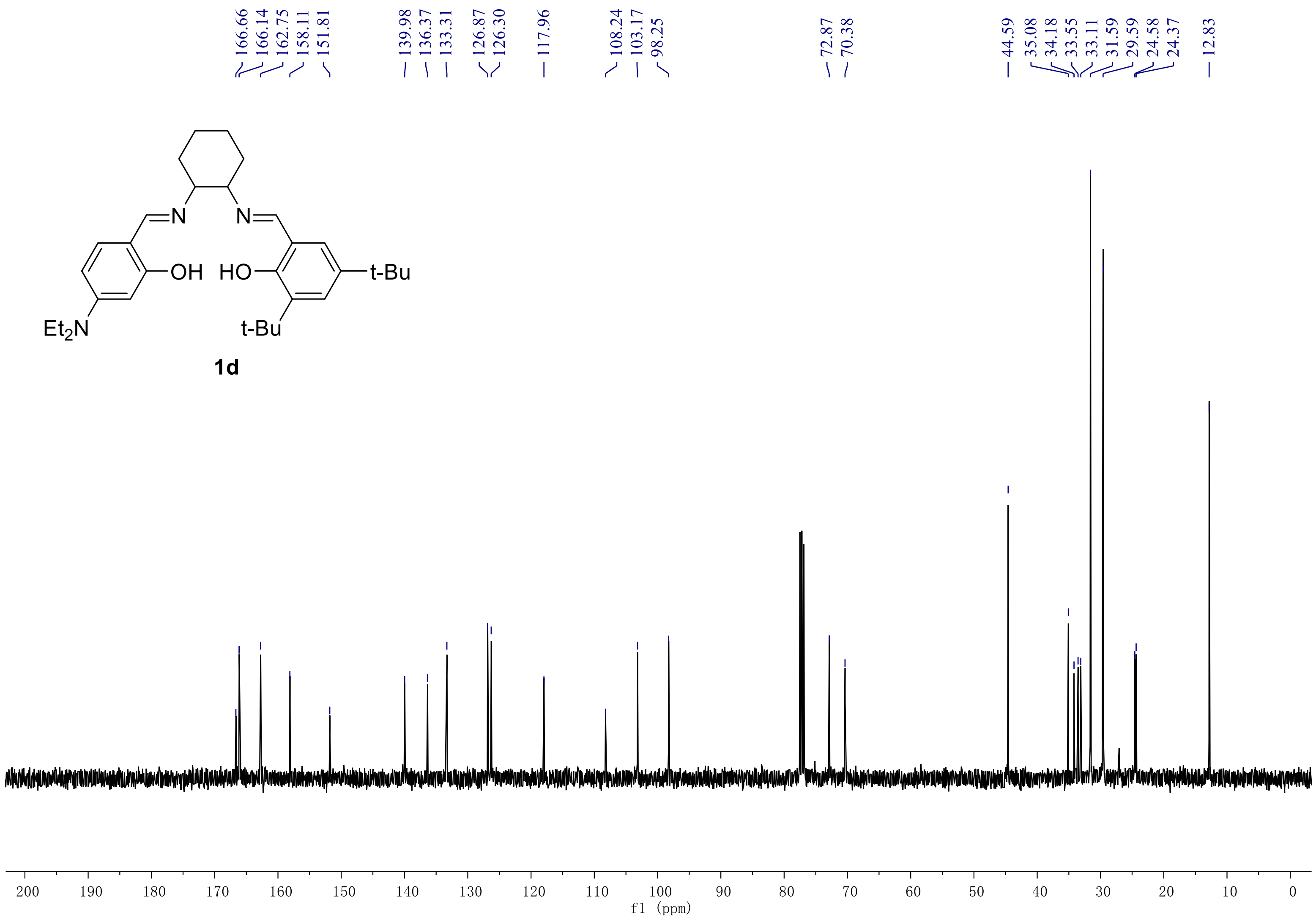
166.66
166.14
162.75
158.11
151.81
139.98
136.37
133.31
126.87
126.30
117.96
108.24
103.17
98.25
72.87
70.38
44.59
35.08
34.18
33.55
33.11
31.59
29.59
24.58
24.37
12.83
OH
HO
t-Bu
Et2N
N
1d
200 190 180 170 160 150 140 130 120 110 100 90 80 70 60 50 40 30 20 10 0
f1 (ppm)

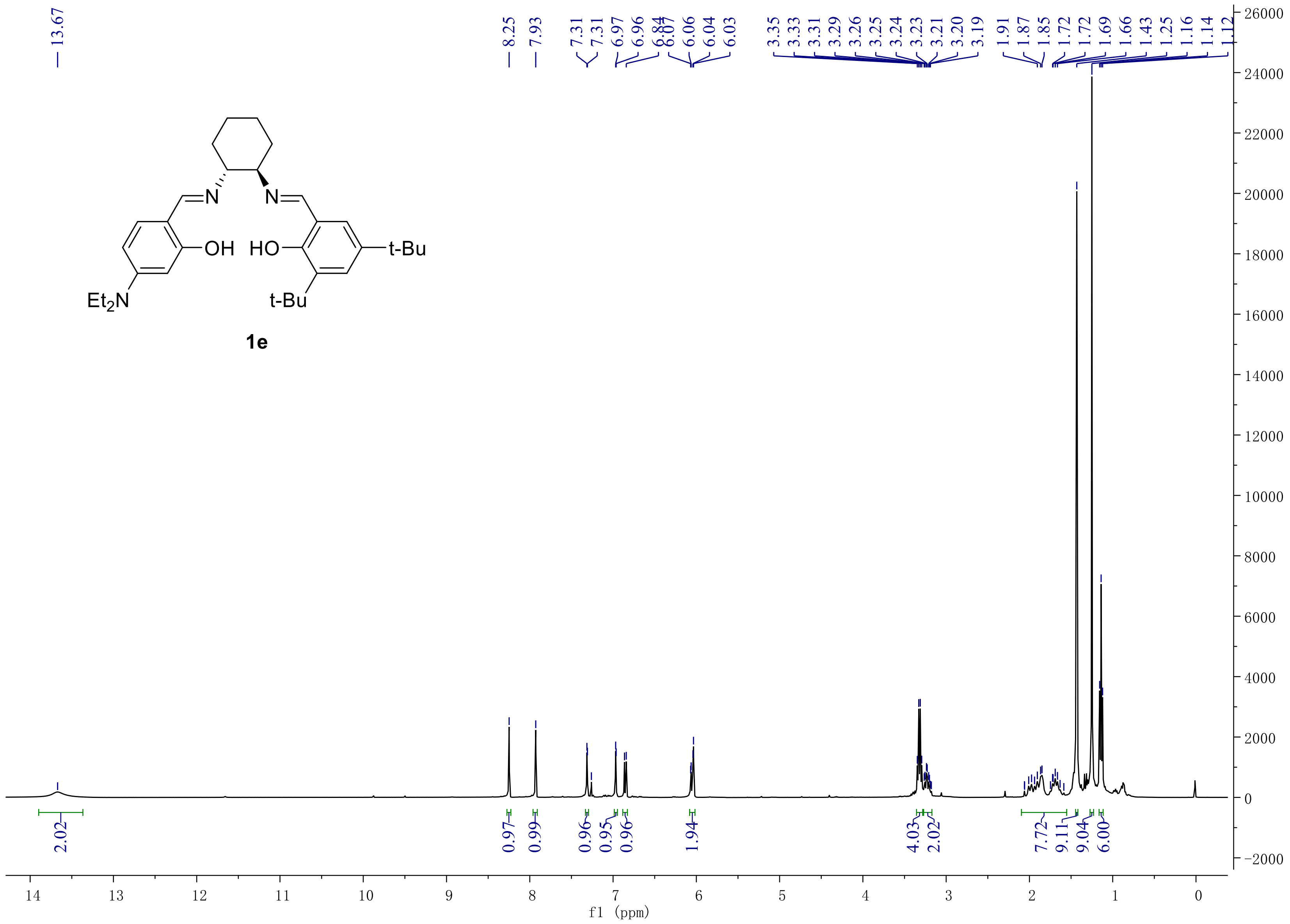

13.67
8.25
7.93
7.31
7.31
6.97
6.96
6.84
6.07
6.06
6.04
6.03
3.35
3.33
3.31
3.29
3.26
3.25
3.24
3.23
3.21
3.20
3.19
1.91
1.87
1.85
1.72
1.72
1.69
1.66
1.43
1.25
1.16
1.14
1.12
OH
HO
N
N
t-Bu
t-Bu
Et2N
1e
2.02
0.97
0.99
0.96
0.95
0.96
1.94
4.03
2.02
7.72
9.11
9.04
6.00
f1 (ppm)

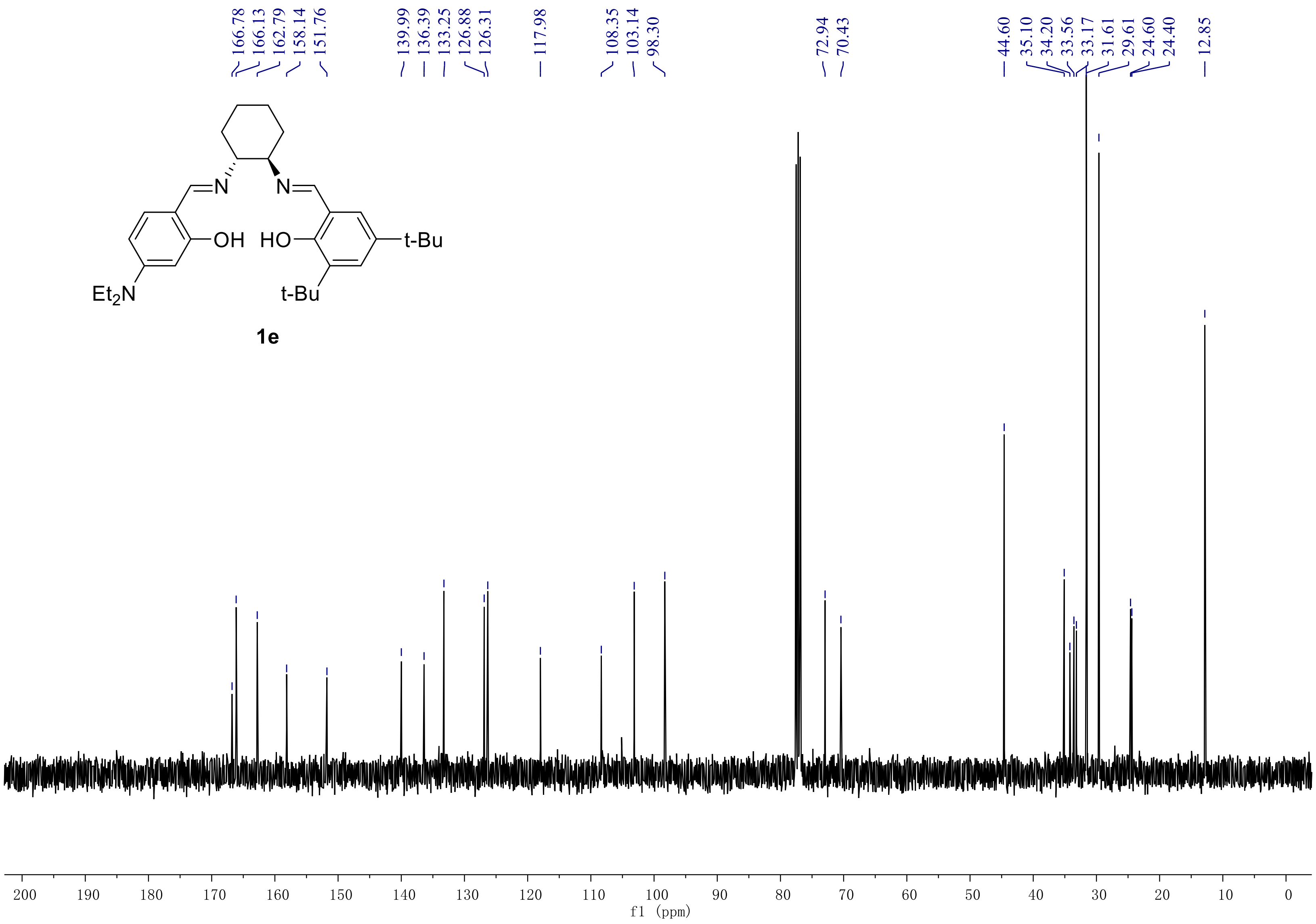
166.78
166.13
162.79
158.14
151.76
139.99
136.39
133.25
126.88
126.31
117.98
108.35
103.14
98.30
72.94
70.43
44.60
35.10
34.20
33.56
33.17
31.61
29.61
24.60
24.40
12.85
OH
HO
t-Bu
t-Bu
Et_2N
N
N
1e
200
190
180
170
160
150
140
130
120
110
100
90
80
70
60
50
40
30
20
10
0
f1 (ppm)

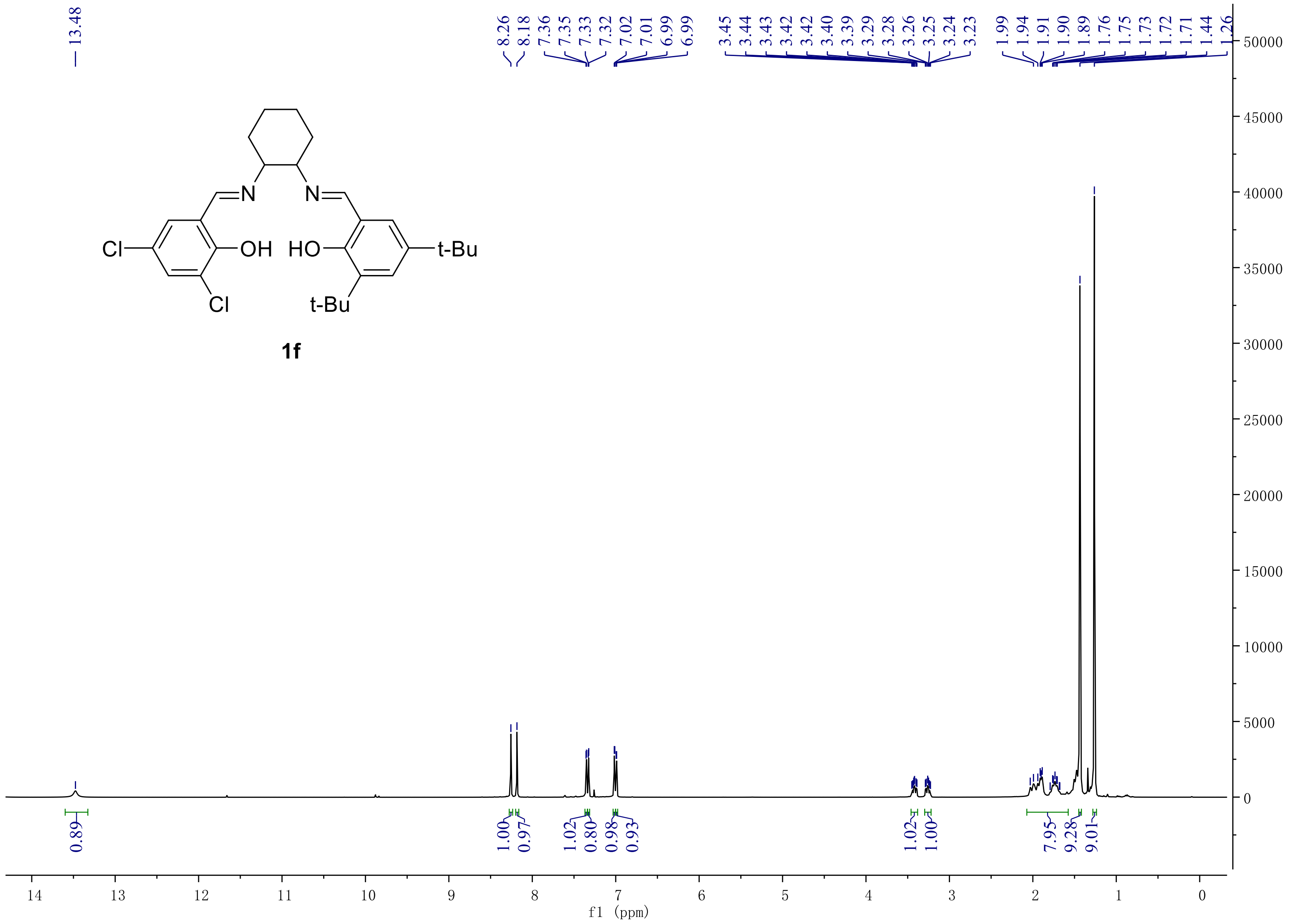

13.48
8.26
8.18
7.36
7.35
7.33
7.32
7.02
7.01
6.99
6.99
3.45
3.44
3.43
3.42
3.42
3.40
3.39
3.29
3.28
3.26
3.25
3.24
3.23
1.99
1.94
1.91
1.90
1.89
1.76
1.75
1.73
1.72
1.71
1.44
1.26
Cl
OH
HO
t-Bu
Cl
t-Bu
1f
0.89
1.00
0.97
1.02
0.80
0.98
0.93
1.02
1.00
7.95
9.28
9.01
f1 (ppm)

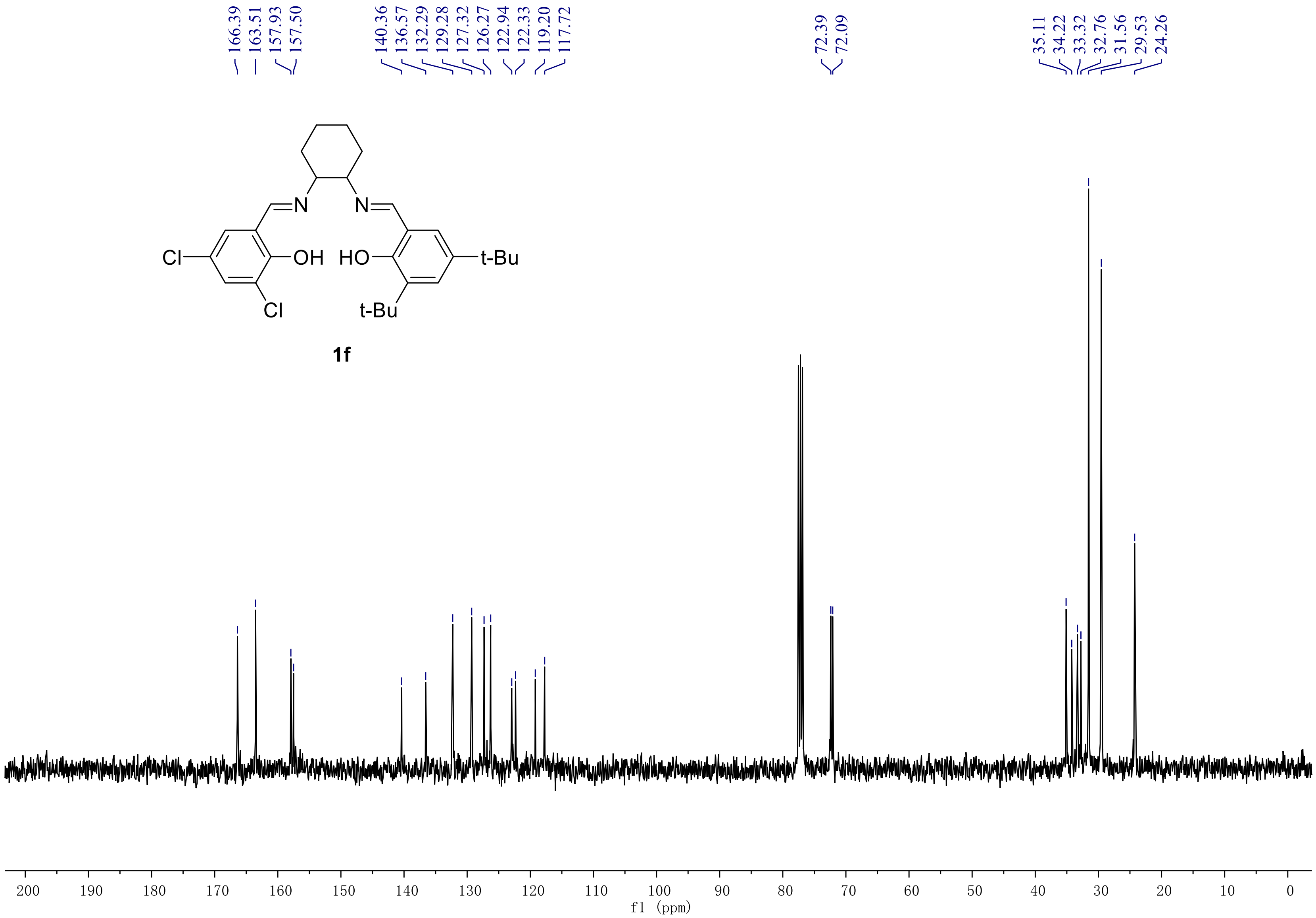
166.39
163.51
157.93
157.50
140.36
136.57
132.29
129.28
127.32
126.27
122.94
122.33
119.20
117.72
72.39
72.09
35.11
34.22
33.32
32.76
31.56
29.53
24.26
Cl
OH
HO
t-Bu
Cl
t-Bu
N
N
1f
200
190
180
170
160
150
140
130
120
110
100
90
80
70
60
50
40
30
20
10
0
f1 (ppm)

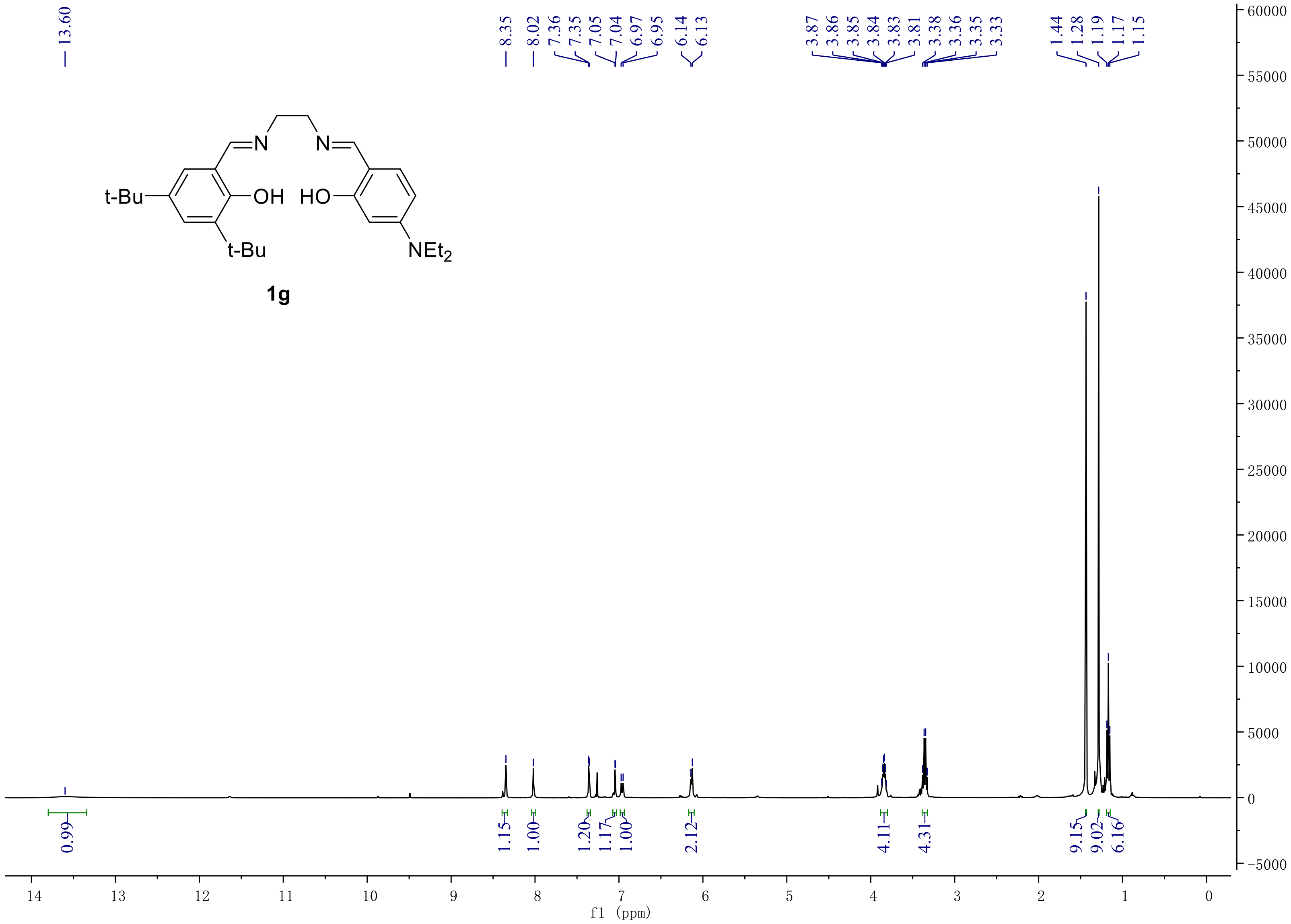

13.60
8.35
8.02
7.36
7.35
7.05
7.04
6.97
6.95
6.14
6.13
3.87
3.86
3.85
3.84
3.83
3.81
3.38
3.36
3.35
3.33
1.44
1.28
1.19
1.17
1.15
t-Bu
OH
HO
t-Bu
NEt2
1g
0.99
1.15
1.00
1.20
1.17
1.00
2.12
4.11
4.31
9.15
9.02
6.16
f1 (ppm)

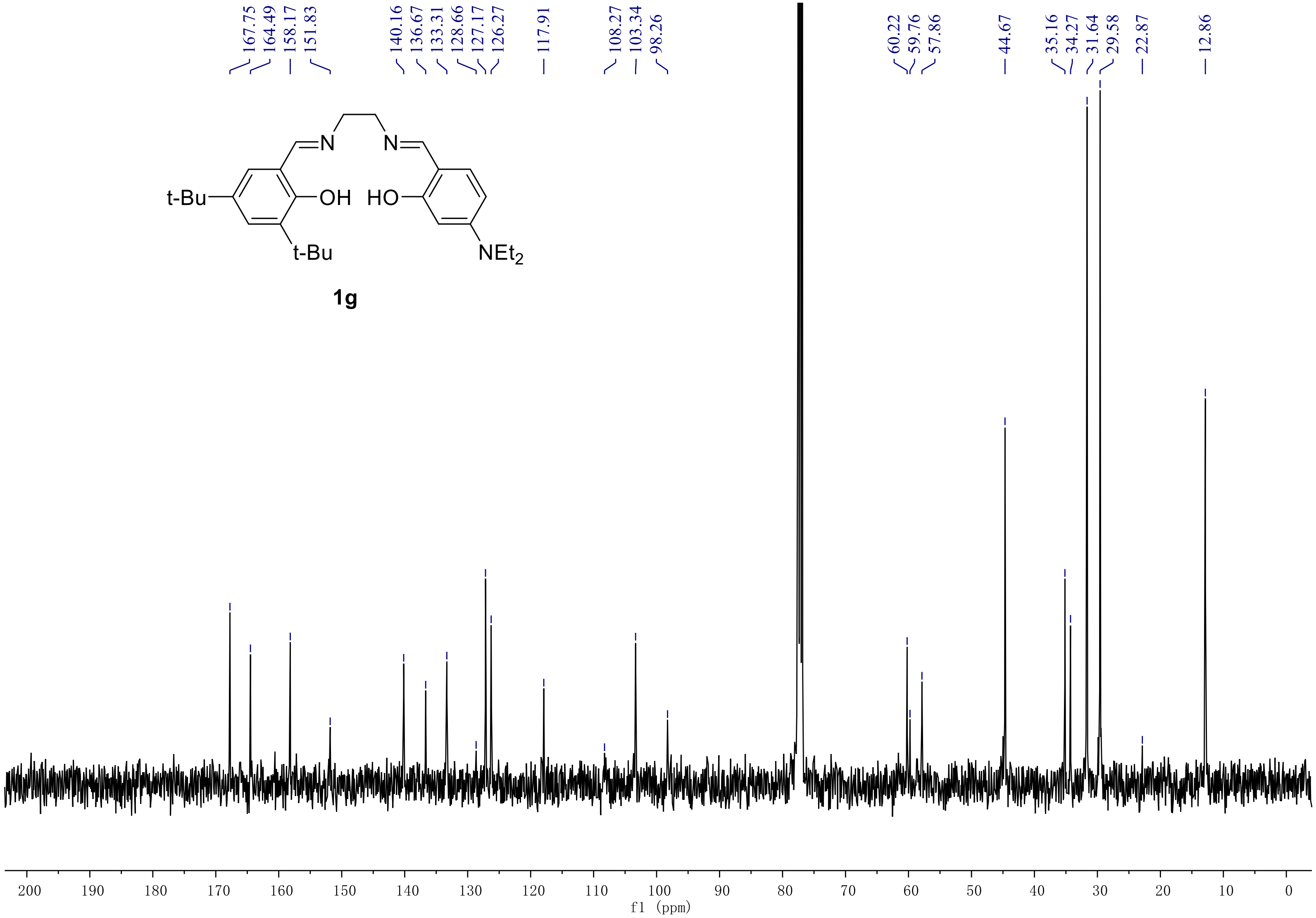

167.75
164.49
158.17
151.83
140.16
136.67
133.31
128.66
127.17
126.27
117.91
108.27
103.34
98.26
60.22
59.76
57.86
44.67
35.16
34.27
31.64
29.58
22.87
12.86
t-Bu
OH
HO
t-Bu
NEt2
1g
200
190
180
170
160
150
140
130
120
110
100
90
80
70
60
50
40
30
20
10
0
f1 (ppm)

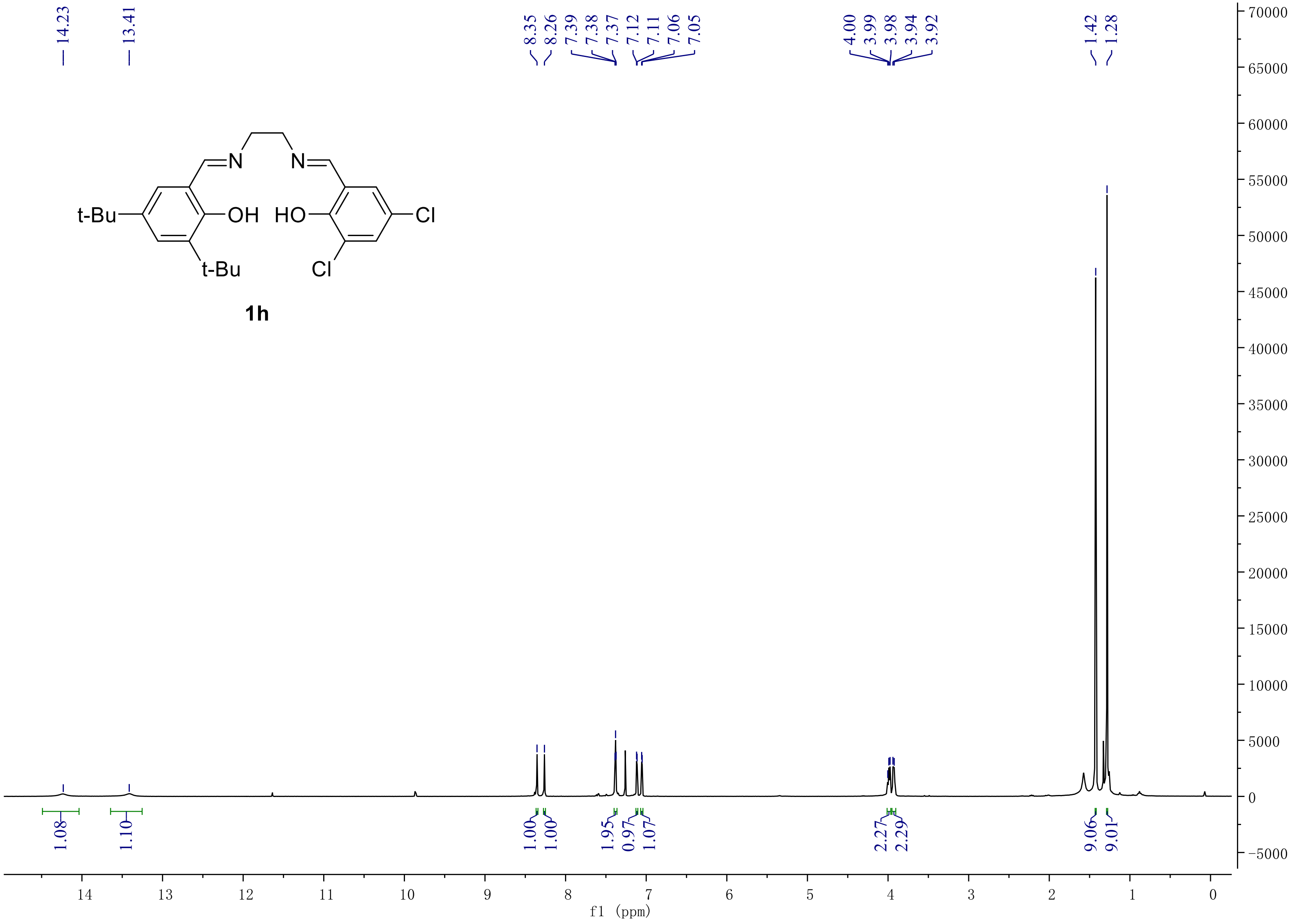

14.23
13.41
8.35
8.26
7.39
7.38
7.37
7.12
7.11
7.06
7.05
4.00
3.99
3.98
3.94
3.92
1.42
1.28
t-Bu
OH
HO
Cl
N
t-Bu
Cl
1h
1.08
1.10
1.00
1.00
1.95
0.97
1.07
2.27
2.29
9.06
9.01
f1 (ppm)

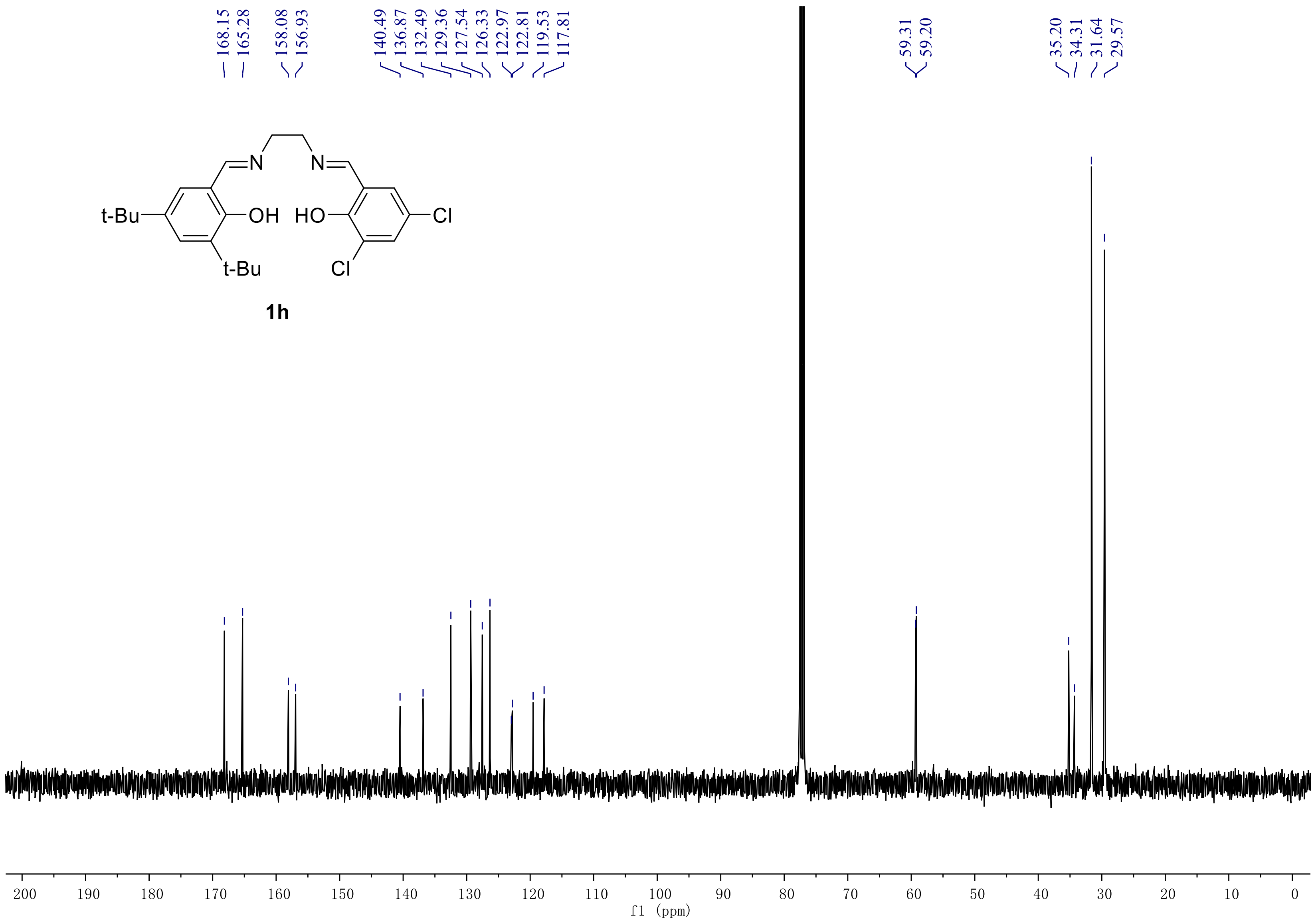

168.15
165.28
158.08
156.93
140.49
136.87
132.49
129.36
127.54
126.33
122.97
122.81
119.53
117.81
59.31
59.20
35.20
34.31
31.64
29.57
N
N
t-Bu
OH
HO
Cl
t-Bu
Cl
1h
200 190 180 170 160 150 140 130 120 110 100 90 80 70 60 50 40 30 20 10 0
f1 (ppm)

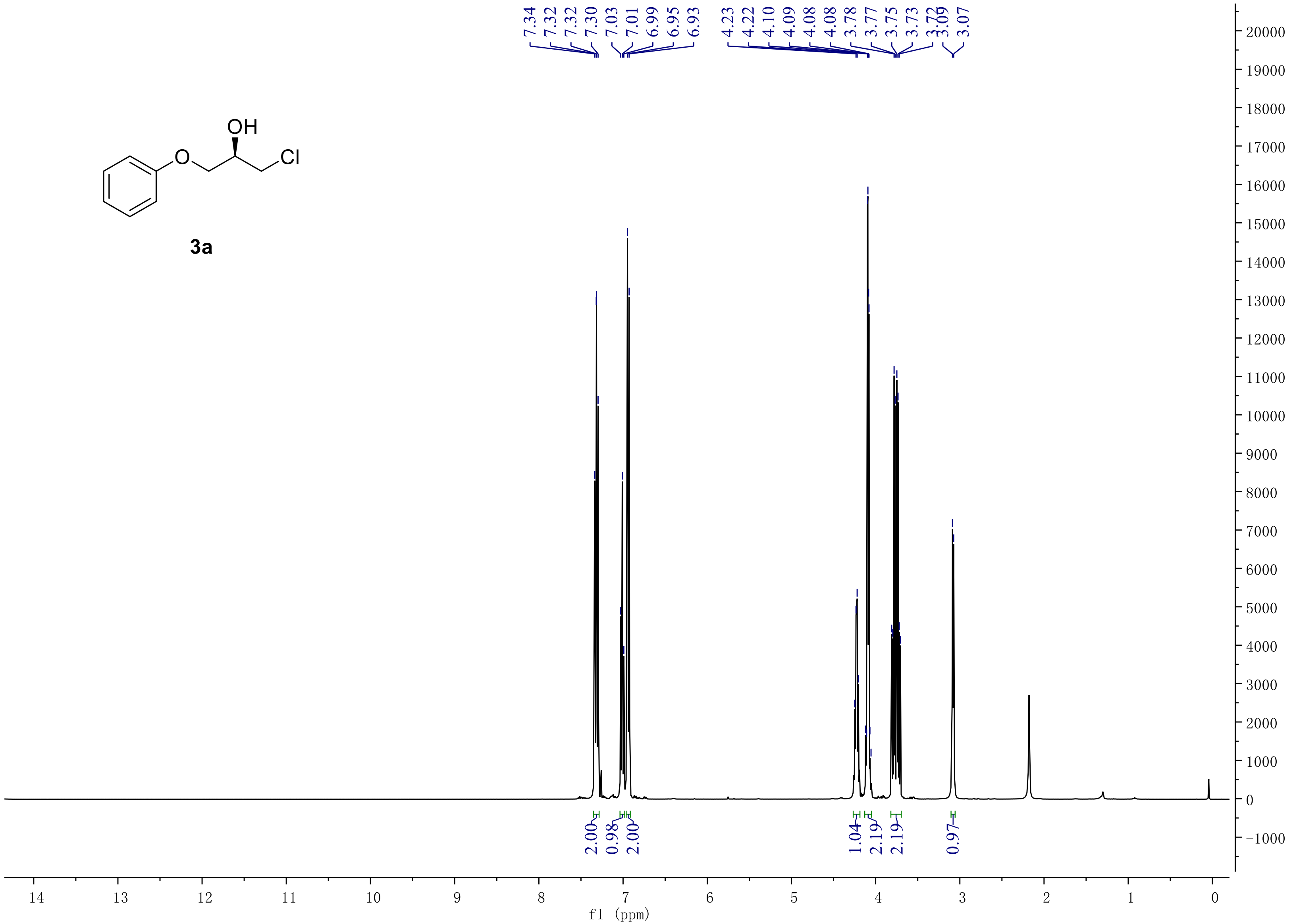
OH
O
Cl
3a
7.34
7.32
7.32
7.30
7.03
7.01
6.99
6.95
6.93
4.23
4.22
4.10
4.09
4.08
4.08
3.78
3.77
3.75
3.73
3.72
3.09
3.07
2.00
0.98
2.00
1.04
2.19
2.19
0.97
f1 (ppm)

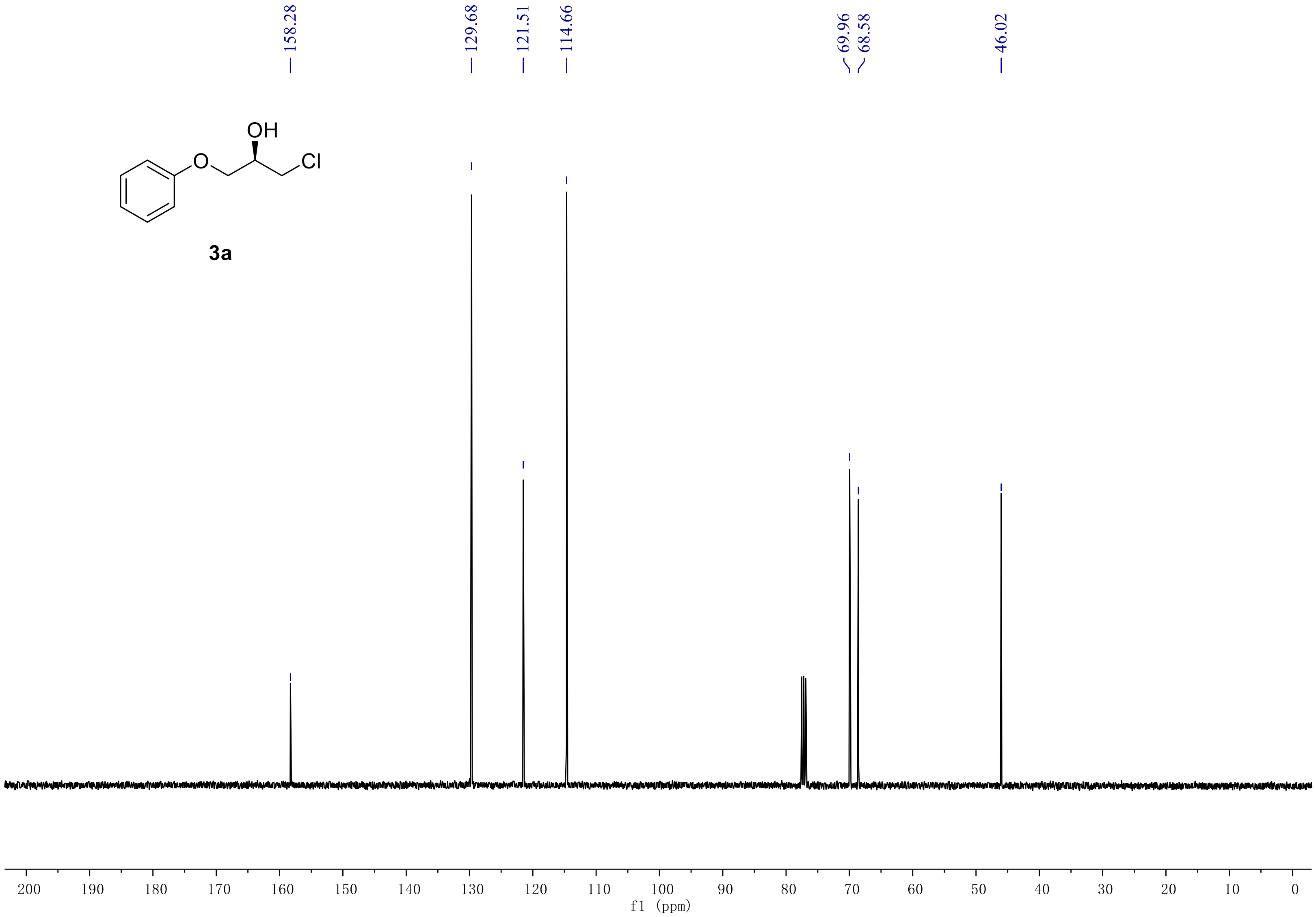
158.28
129.68
121.51
114.66
69.96
68.58
46.02
OH
O
Cl
3a
200 190 180 170 160 150 140 130 120 110 100 90 80 70 60 50 40 30 20 10 0
f1 (ppm)

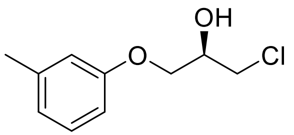
OH
O
Cl

3b

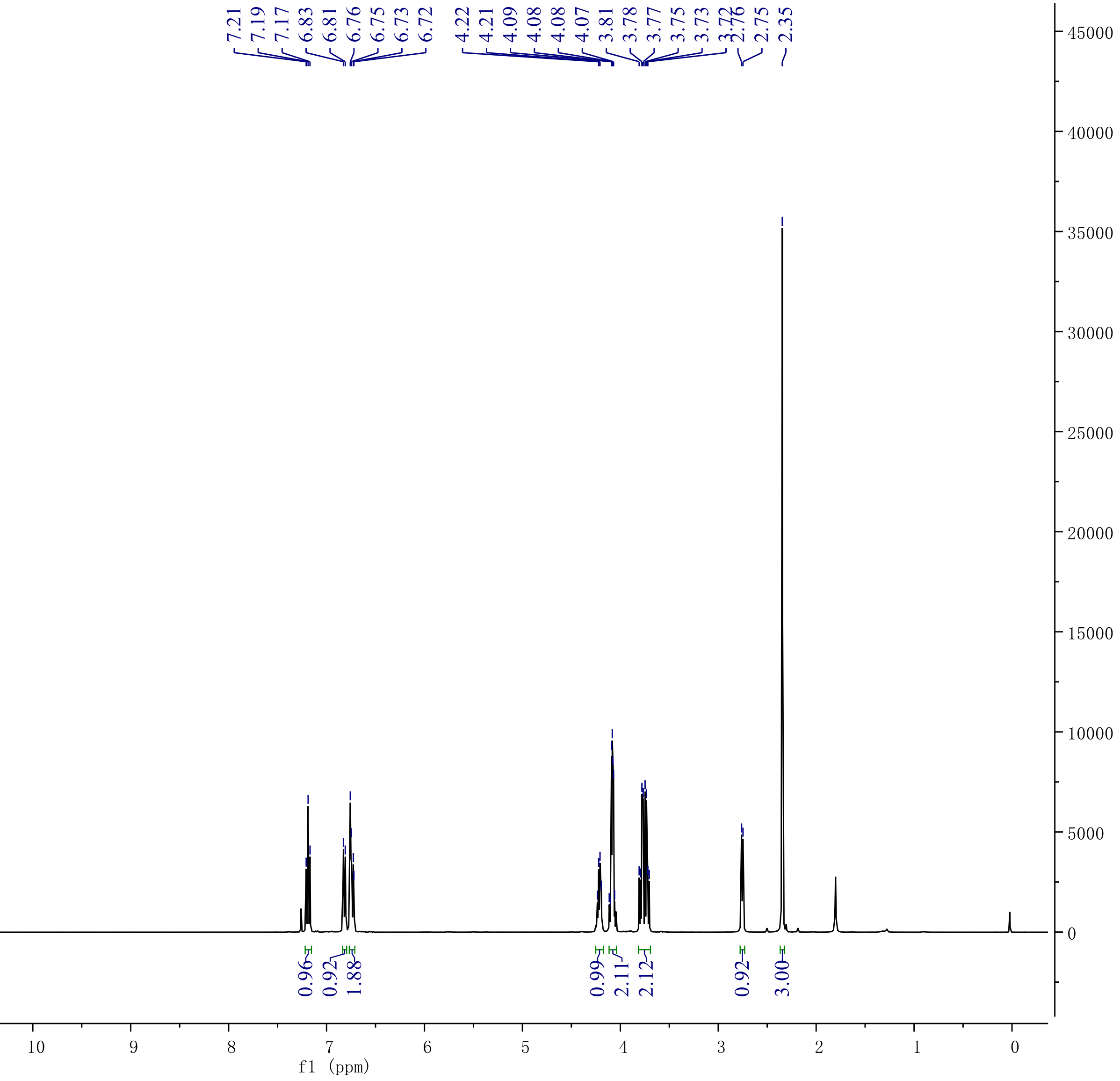
7.21
7.19
7.17
6.83
6.81
6.76
6.75
6.73
6.72
4.22
4.21
4.09
4.08
4.08
4.07
3.81
3.78
3.77
3.75
3.73
3.72
2.76
2.75
2.35
0.96
0.92
1.88
0.99
2.11
2.12
0.92
3.00
45000
40000
35000
30000
25000
20000
15000
10000
5000
0
14 13 12 11 10 9 8 7 6 5 4 3 2 1 0
f1 (ppm)

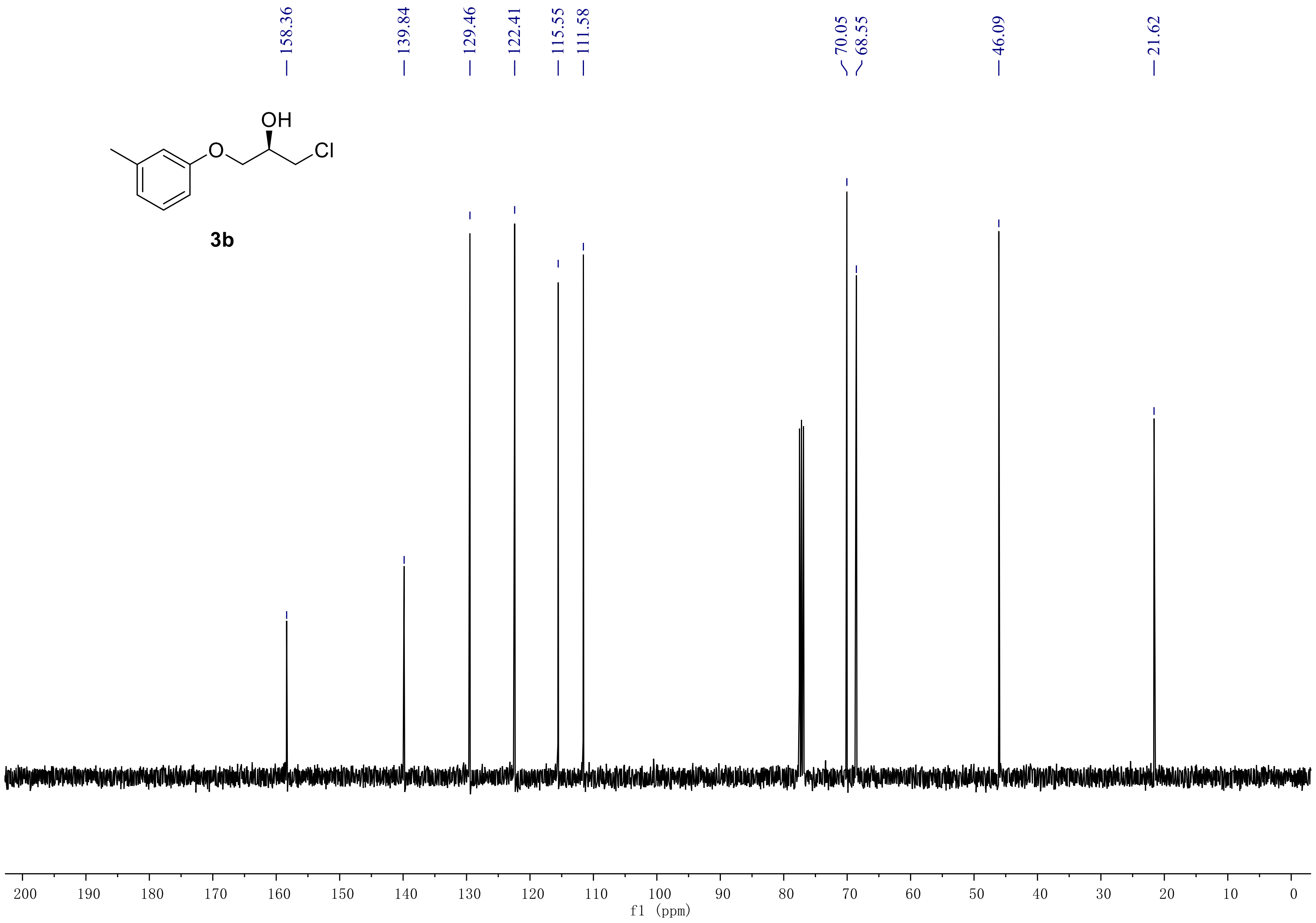

158.36
139.84
129.46
122.41
115.55
111.58
70.05
68.55
46.09
21.62
OH
O
Cl
3b
200 190 180 170 160 150 140 130 120 110 100 90 80 70 60 50 40 30 20 10 0
f1 (ppm)

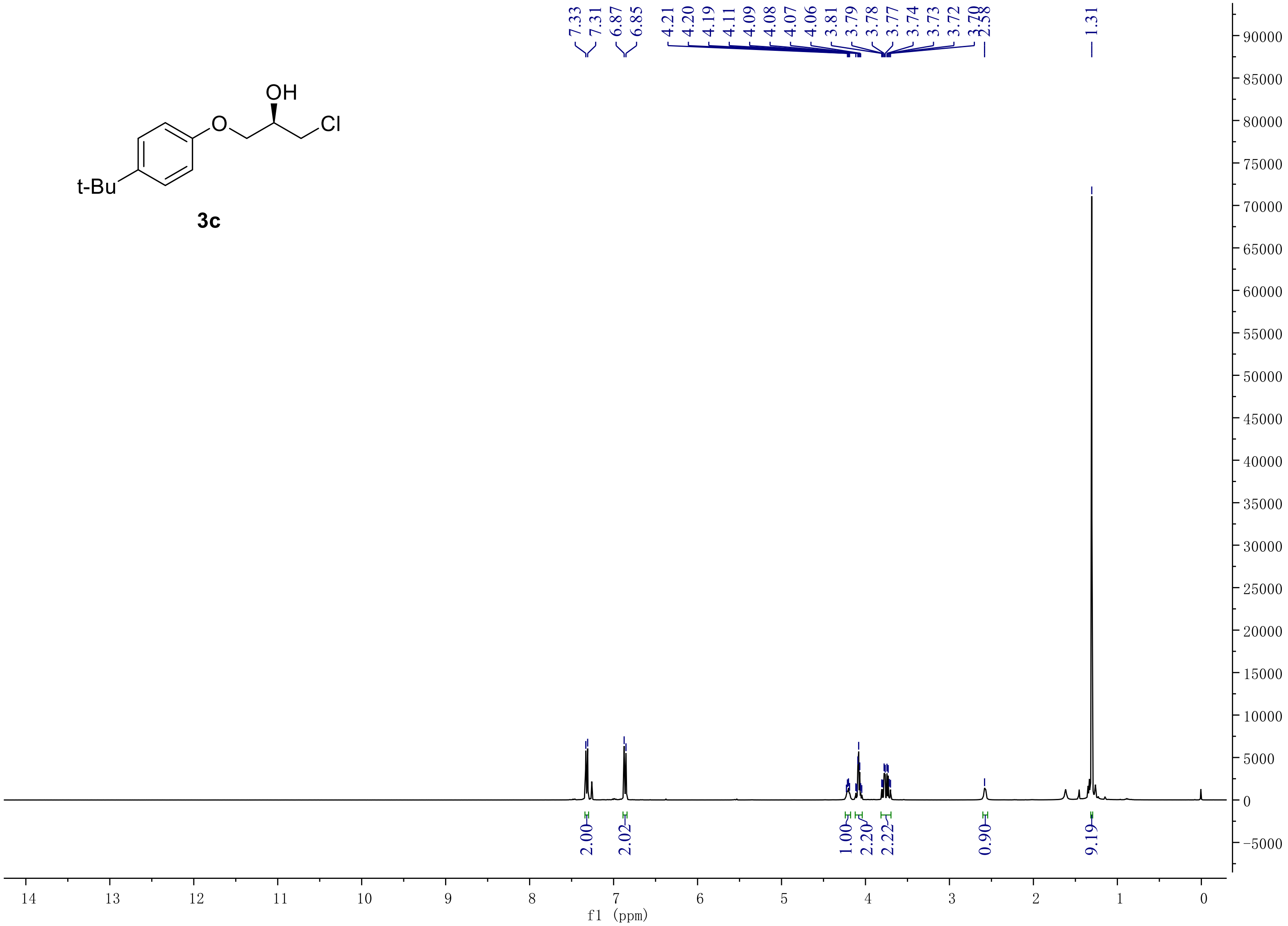
OH
O
Cl
t-Bu
3c
7.33
7.31
6.87
6.85
4.21
4.20
4.19
4.11
4.09
4.08
4.07
4.06
3.81
3.79
3.78
3.77
3.74
3.73
3.72
3.70
2.58
1.31
2.00
2.02
1.00
2.20
2.22
0.90
9.19
f1 (ppm)

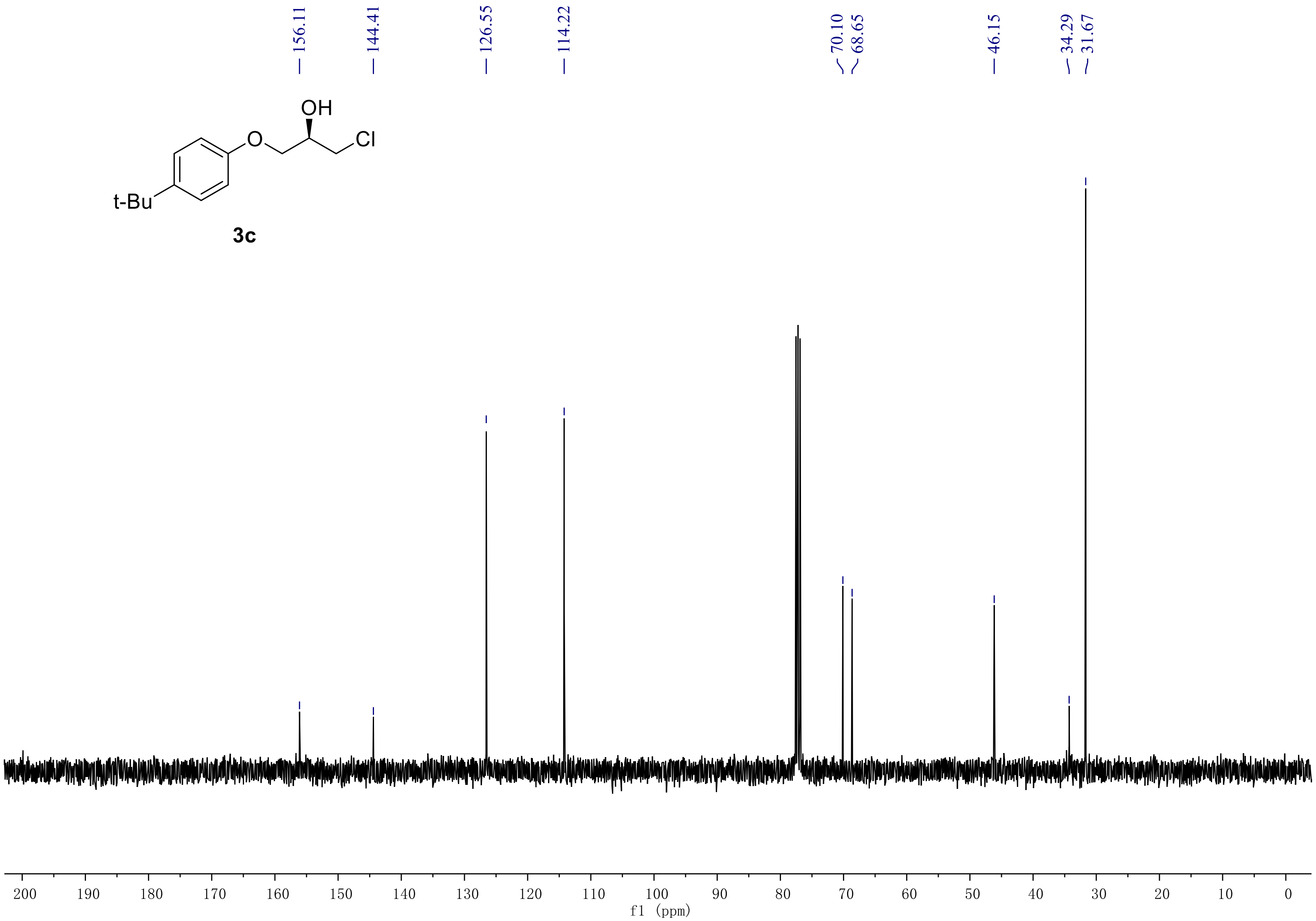
156.11
144.41
126.55
114.22
70.10
68.65
46.15
34.29
31.67
OH
O
Cl
t-Bu
3c
200 190 180 170 160 150 140 130 120 110 100 90 80 70 60 50 40 30 20 10 0
f1 (ppm)

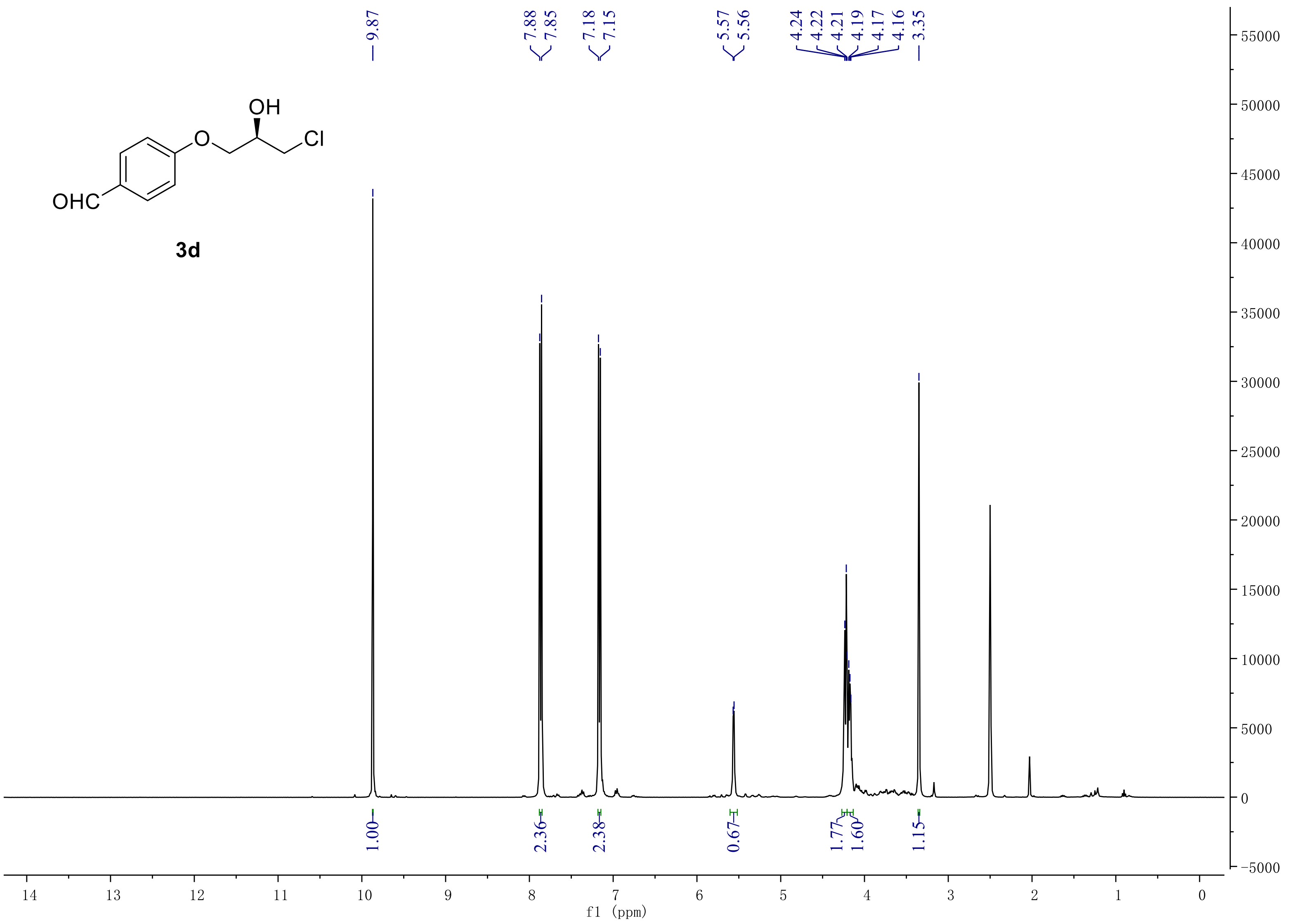

OH
O
Cl
OHC
3d
9.87
7.88
7.85
7.18
7.15
5.57
5.56
4.24
4.22
4.21
4.19
4.17
4.16
3.35
1.00
2.36
2.38
0.67
1.77
1.60
1.15
f1 (ppm)

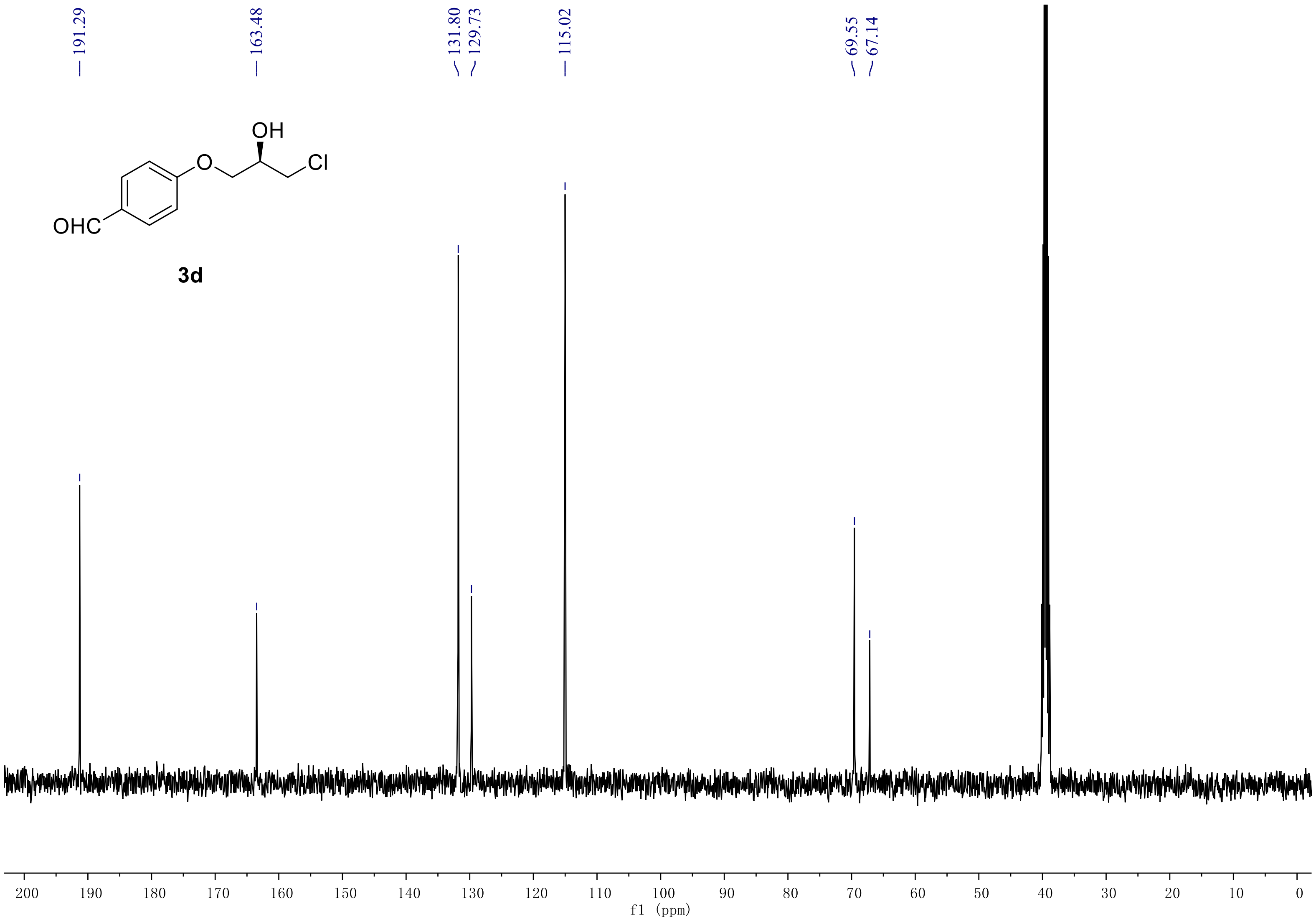
191.29
163.48
131.80
129.73
115.02
69.55
67.14
OH
O
Cl
OHC
3d
200 190 180 170 160 150 140 130 120 110 100 90 80 70 60 50 40 30 20 10 0
f1 (ppm)

IR spectra

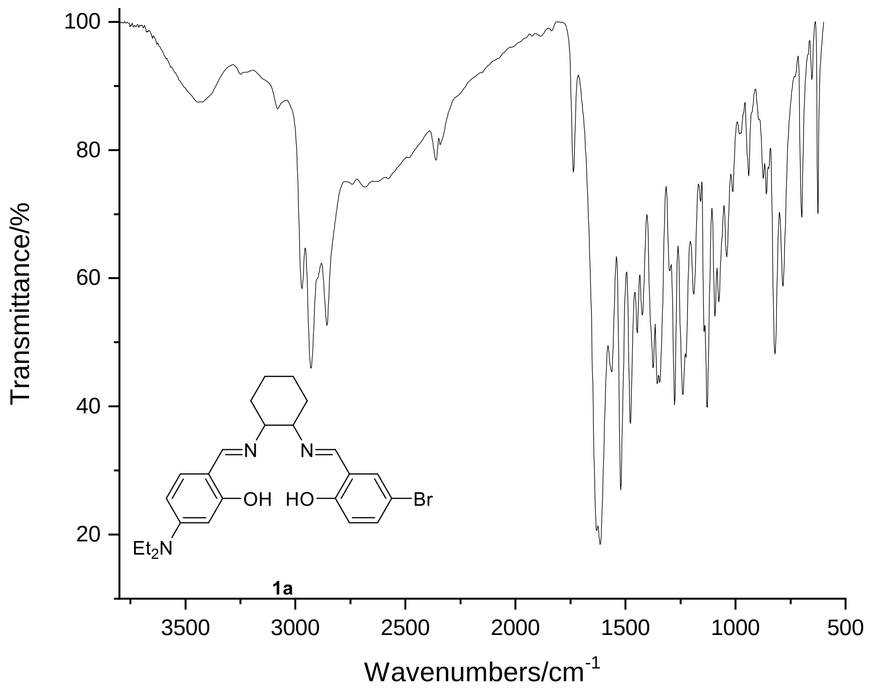

Transmittance/%
100
80
60
40
20
3500
3000
2500
2000
1500
1000
500
Wavenumbers/cm-1
N
N
OH
HO
Br
Et2N
1a

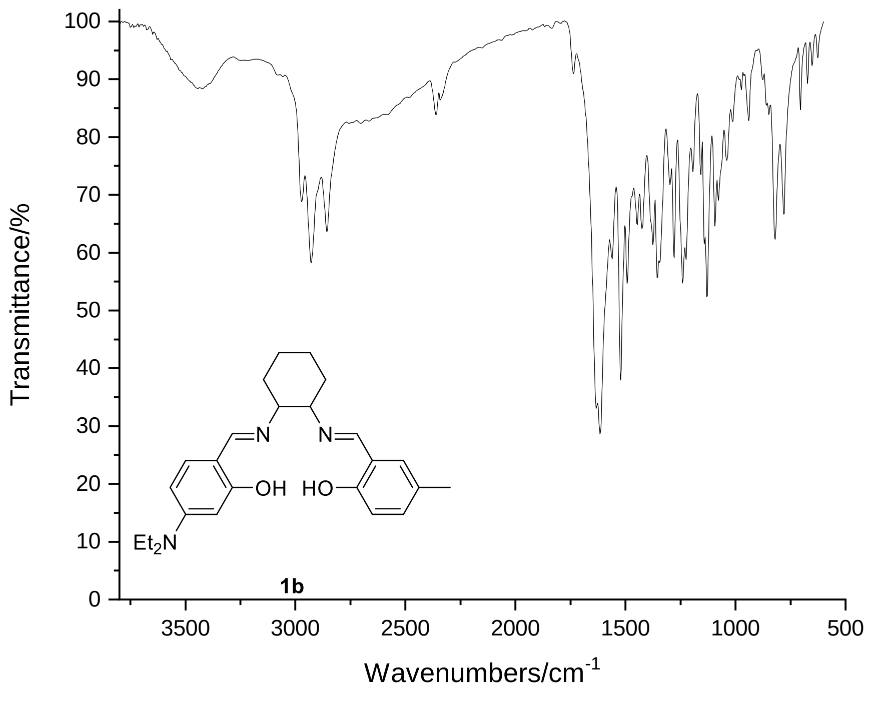

Transmittance/%
100
90
80
70
60
50
40
30
20
10
0
3500
3000
2500
2000
1500
1000
500
Wavenumbers/cm-1
N
N
OH
HO
Et2N
1b

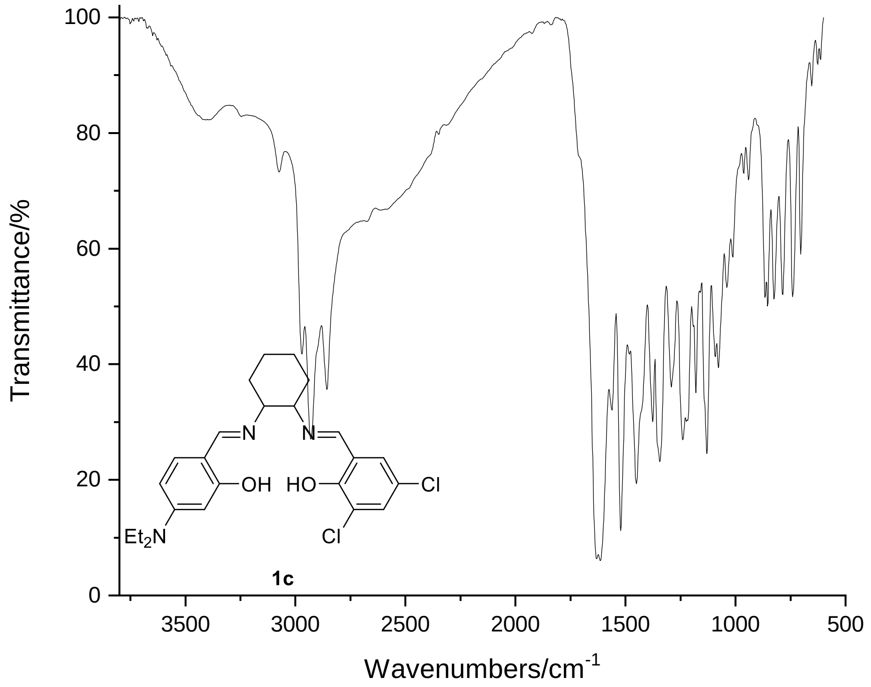
Transmittance/%
100
80
60
40
20
0
3500
3000
2500
2000
1500
1000
500
Wavenumbers/cm-1
N
N
OH
HO
Cl
Cl
Et2N
1c

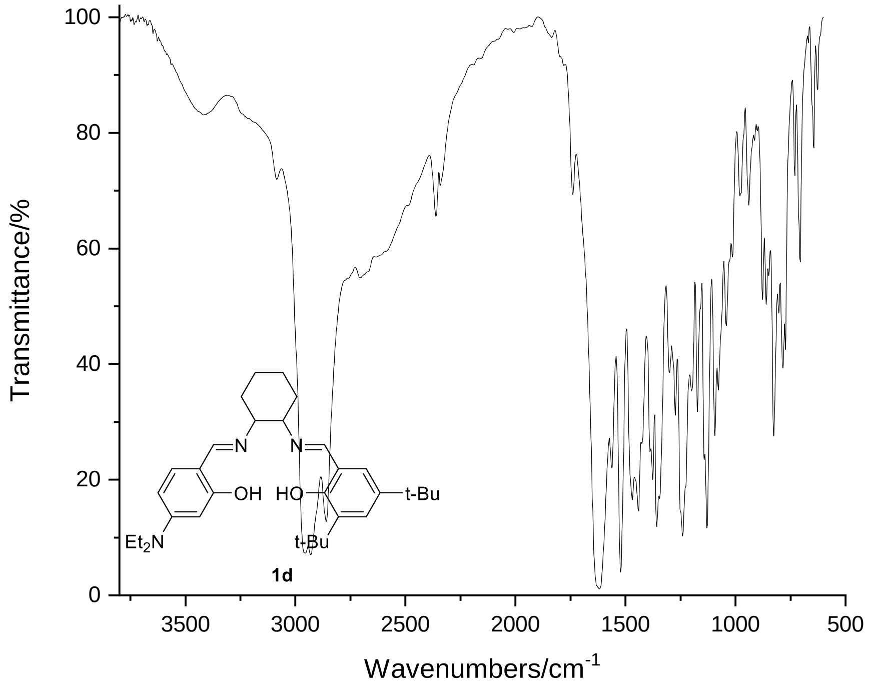
Transmittance/%
100
80
60
40
20
0
3500
3000
2500
2000
1500
1000
500
Wavenumbers/cm-1
N
N
OH
HO
t-Bu
t-Bu
Et2N
1d

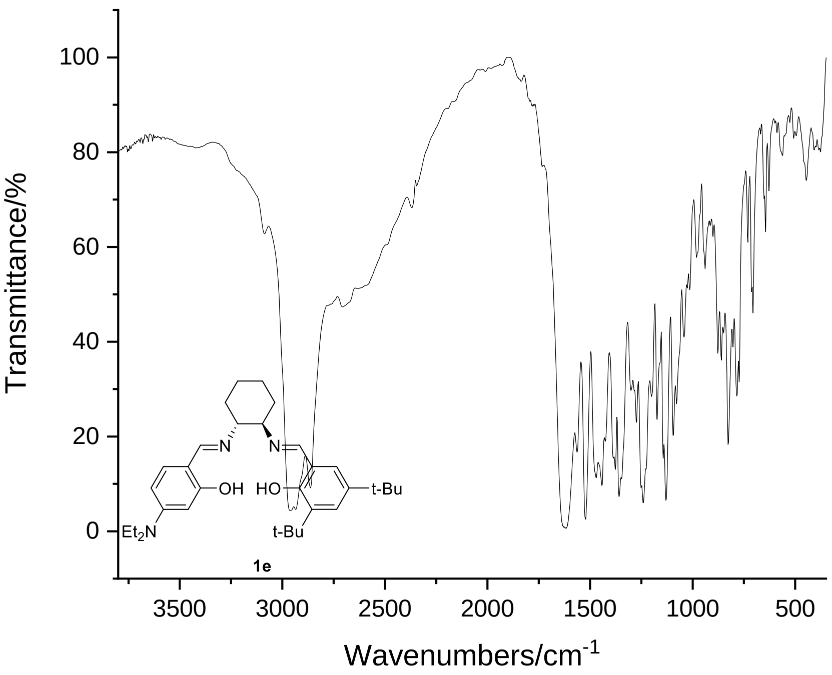
Transmittance/%
Wavenumbers/cm-1
100
80
60
40
20
0
3500
3000
2500
2000
1500
1000
500
Et2N
OH
HO
t-Bu
t-Bu
N
N
1e

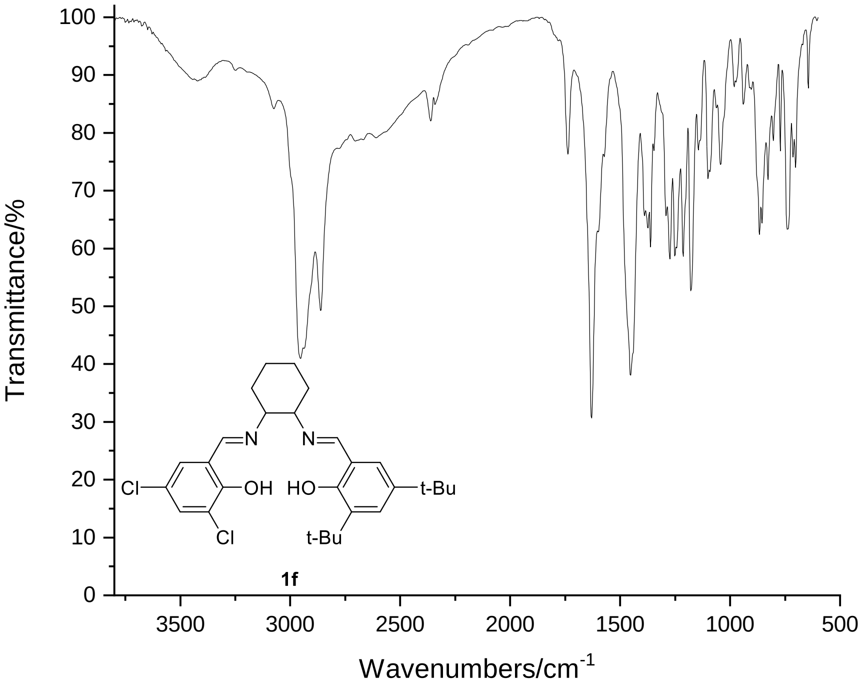
Transmittance/%
Wavenumbers/cm-1
100
90
80
70
60
50
40
30
20
10
0
3500
3000
2500
2000
1500
1000
500
Cl
Cl
OH
HO
t-Bu
t-Bu
N
N
1f

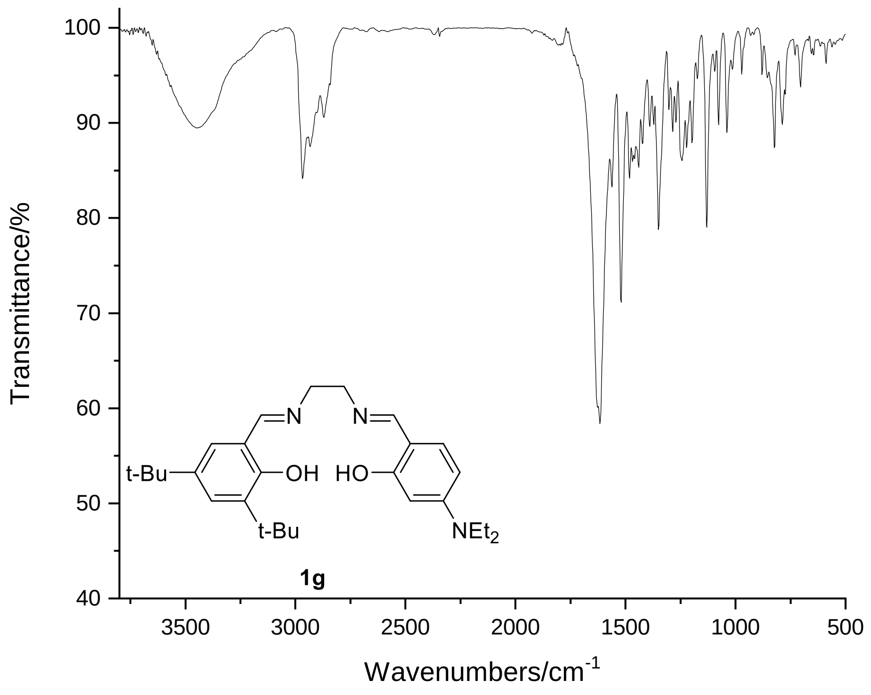
Transmittance/%
100
90
80
70
60
50
40
3500
3000
2500
2000
1500
1000
500
Wavenumbers/cm-1
t-Bu
OH
HO
t-Bu
NEt2
N
N
1g

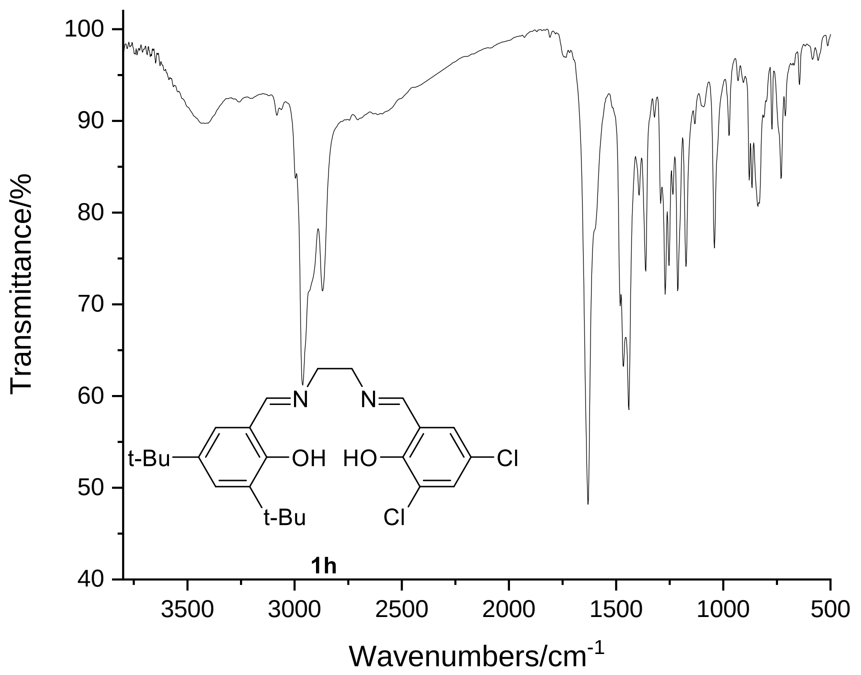
Transmittance/%
100
90
80
70
60
50
40
3500
3000
2500
2000
1500
1000
500
Wavenumbers/cm-1
t-Bu
OH
HO
Cl
t-Bu
Cl
N
N
1h

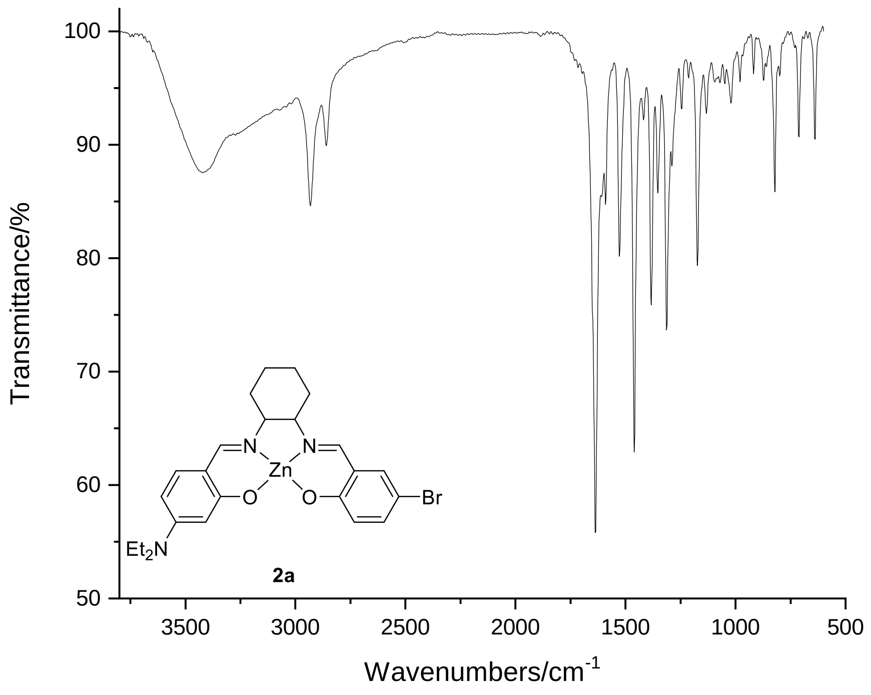
Transmittance/%
100
90
80
70
60
50
3500
3000
2500
2000
1500
1000
500
Wavenumbers/cm-1
N
N
Zn
O
O
Br
Et2N
2a

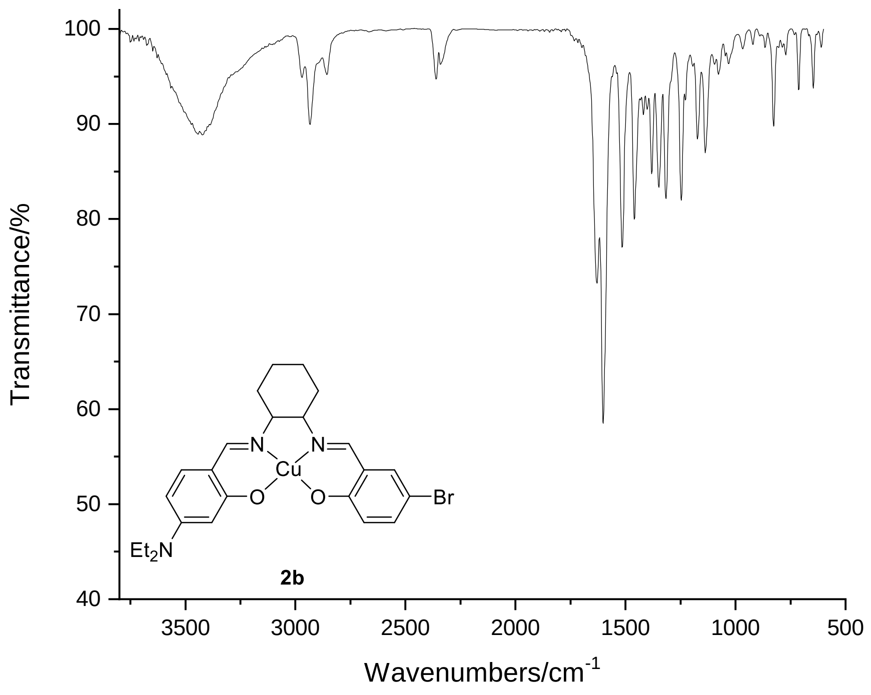
Transmittance/%
100
90
80
70
60
50
40
3500
3000
2500
2000
1500
1000
500
Wavenumbers/cm-1
N
N
Cu
O
O
Br
Et2N
2b

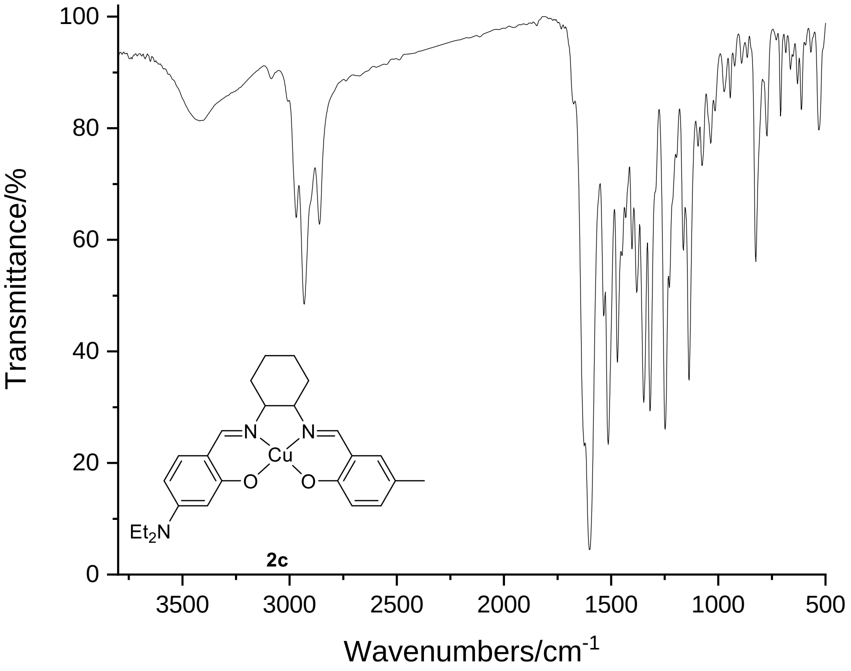
Transmittance/%
Wavenumbers/cm-1
Et2N
Cu
2c

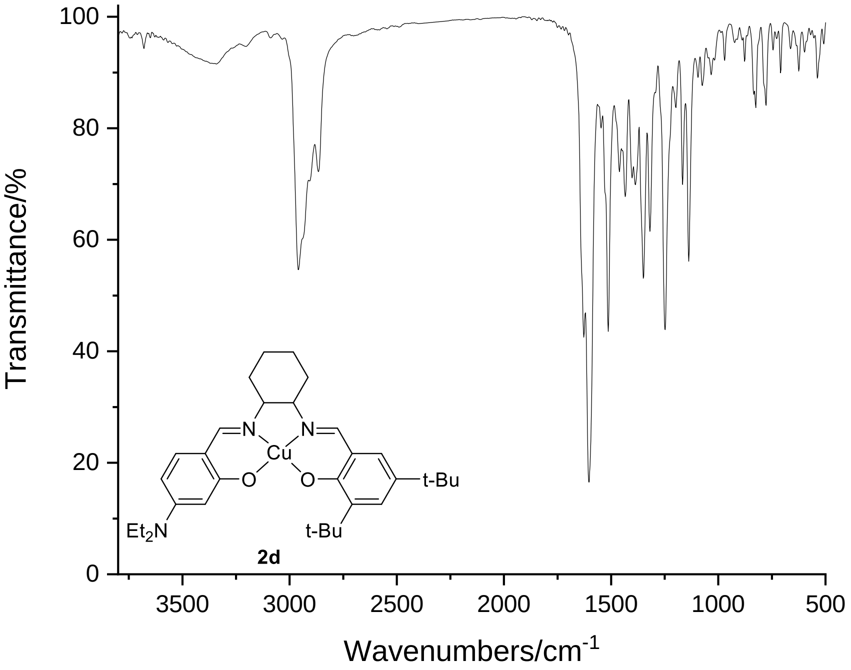
Transmittance/%
Wavenumbers/cm-1
Et2N
Cu
t-Bu
t-Bu
2d

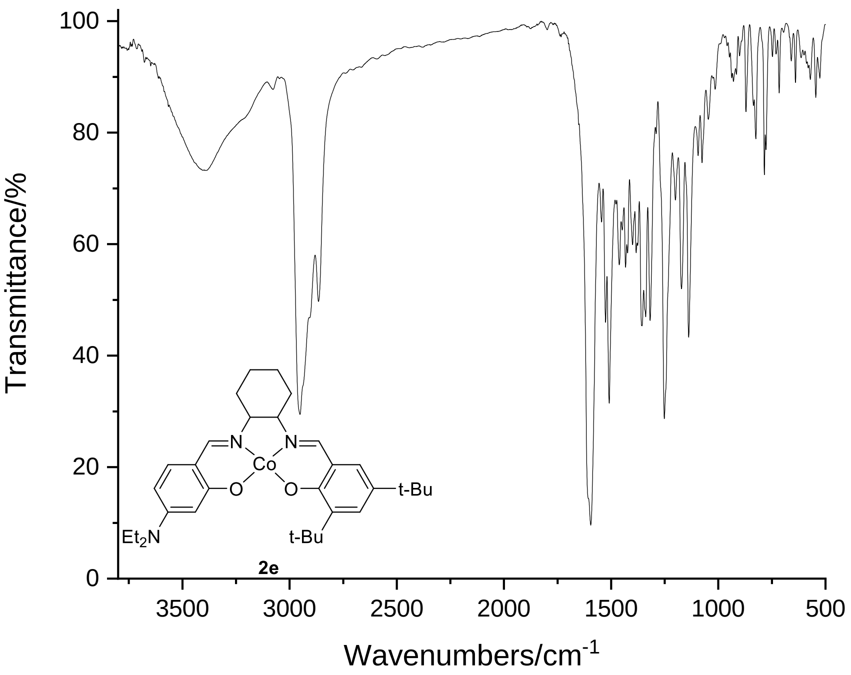
100
80
60
40
20
0
3500
3000
2500
2000
1500
1000
500
Transmittance/%
Wavenumbers/cm-1
Et2N
t-Bu
t-Bu
Co
N
N
O
O
2e

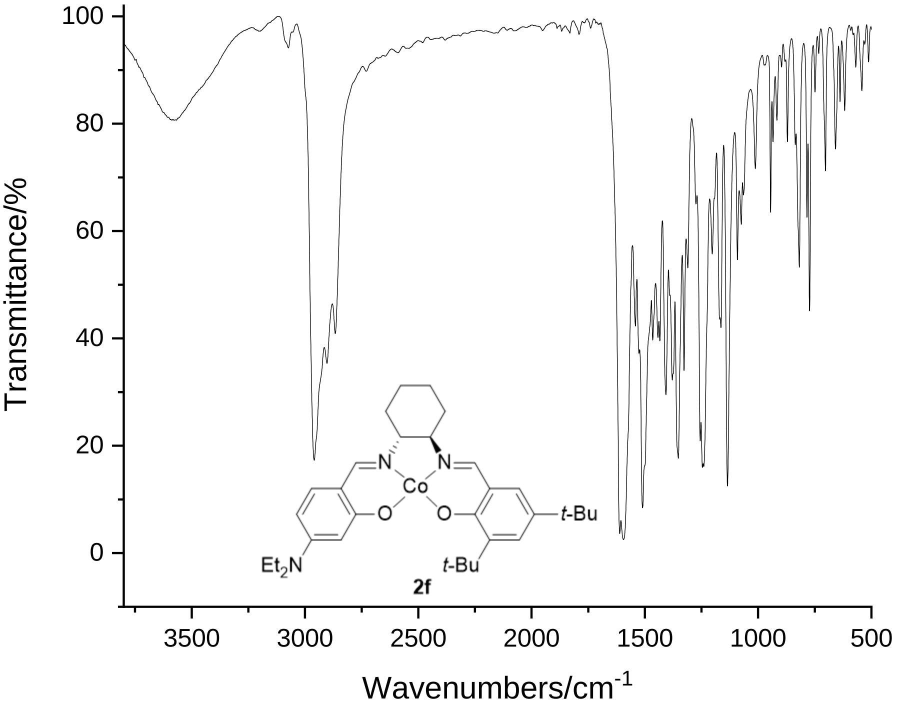
100
80
60
40
20
0
3500
3000
2500
2000
1500
1000
500
Transmittance/%
Wavenumbers/cm-1
Et2N
t-Bu
t-Bu
Co
N
N
O
O
2f

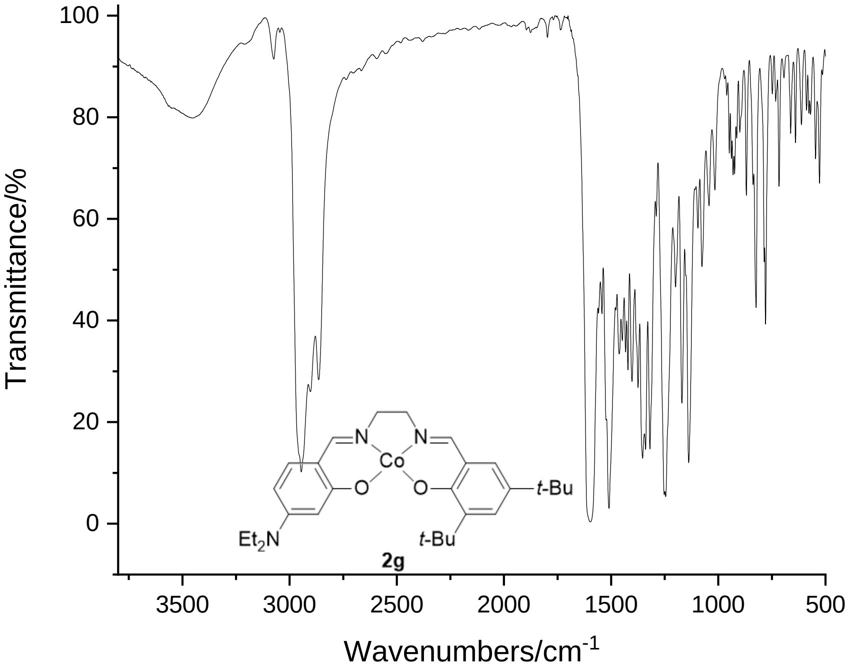

Transmittance/%
100
80
60
40
20
0
3500
3000
2500
2000
1500
1000
500
Wavenumbers/cm-1
N
Co
N
O
O
t-Bu
Et2N
t-Bu
2g

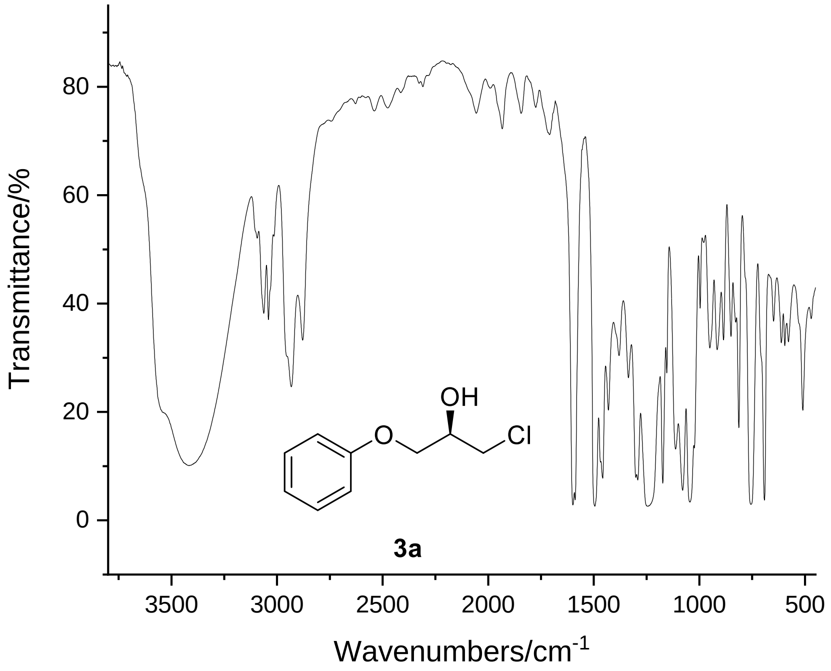

Transmittance/%
Wavenumbers/cm-1
OH
O
Cl
3a

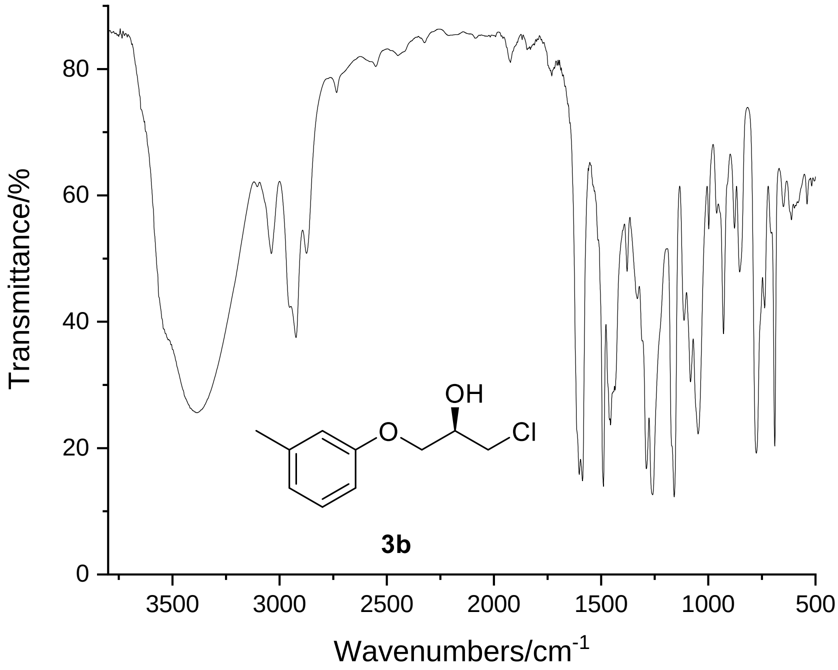

Transmittance/%
Wavenumbers/cm-1
OH
O
Cl
3b

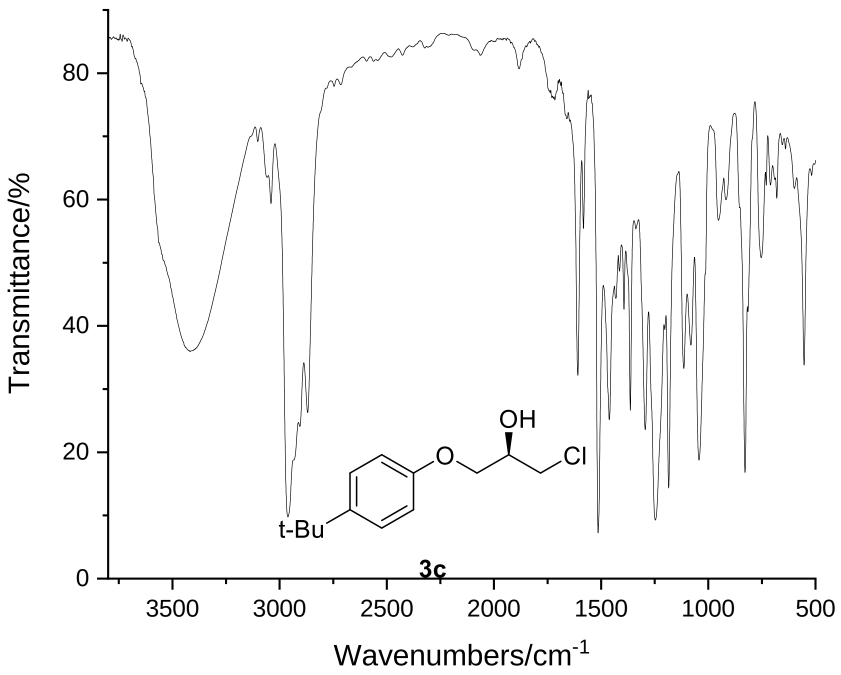
Transmittance/%
0
20
40
60
80
3500
3000
2500
2000
1500
1000
500
Wavenumbers/cm-1
OH
O
Cl
t-Bu
3c

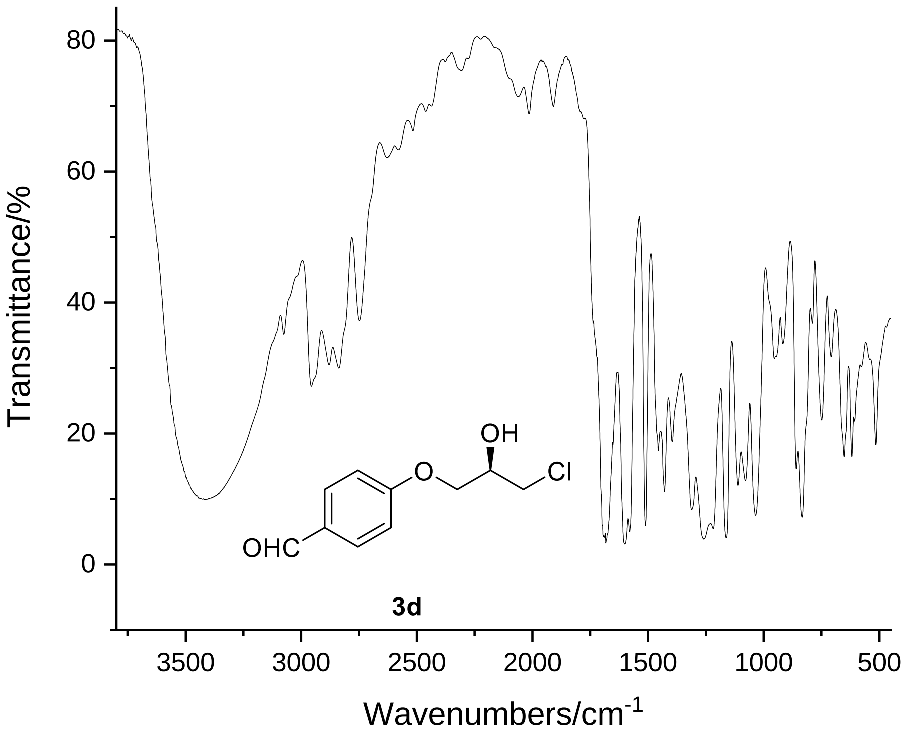
Transmittance/%
0
20
40
60
80
3500
3000
2500
2000
1500
1000
500
Wavenumbers/cm-1
OH
O
Cl
OHC
3d
